# Development of the first *in vivo* GPR17 ligand through an iterative drug discovery pipeline: A novel disease-modifying strategy for multiple sclerosis

**DOI:** 10.1371/journal.pone.0231483

**Published:** 2020-04-22

**Authors:** Chiara Parravicini, Davide Lecca, Davide Marangon, Giusy Tindara Coppolino, Simona Daniele, Elisabetta Bonfanti, Marta Fumagalli, Luca Raveglia, Claudia Martini, Elisabetta Gianazza, Maria Letizia Trincavelli, Maria P. Abbracchio, Ivano Eberini

**Affiliations:** 1 Dipartimento di Scienze Farmacologiche e Biomolecolari, Università degli Studi di Milano, Milan, Italy; 2 Department of Pharmacy, University of Pisa, Pisa, Italy; 3 Aptuit Srl, Verona, Italy; 4 Dipartimento di Scienze Farmacologiche e Biomolecolari & DSRC, Università degli Studi di Milano, Milan, Italy; Instituto Cajal-CSIC, SPAIN

## Abstract

The GPR17 receptor, expressed on oligodendroglial precursors (OPCs, the myelin producing cells), has emerged as an attractive target for a pro-myelinating strategy in multiple sclerosis (MS). However, the proof-of-concept that selective GPR17 ligands actually exert protective activity *in vivo* is still missing. Here, we exploited an iterative drug discovery pipeline to prioritize novel and selective GPR17 pro-myelinating agents out of more than 1,000,000 compounds. We first performed an *in silico* high-throughput screening on GPR17 structural model to identify three chemically-diverse ligand families that were then combinatorially exploded and refined. Top-scoring compounds were sequentially tested on reference pharmacological *in vitro* assays with increasing complexity, ending with myelinating OPC-neuron co-cultures. Successful ligands were filtered through *in silico* simulations of metabolism and pharmacokinetics, to select the most promising hits, whose dose and ability to target the central nervous system were then determined *in vivo*. Finally, we show that, when administered according to a preventive protocol, one of them (named by us as galinex) is able to significantly delay the onset of experimental autoimmune encephalomyelitis (EAE), a mouse model of MS. This outcome validates the predictivity of our pipeline to identify novel MS-modifying agents.

## Introduction

Multiple sclerosis (MS) is a chronic, inflammatory, autoimmune disease of the central nervous system (CNS), characterized by demyelination [1,2]. MS starts as an autoimmune reaction leading to acute CNS inflammation, followed by plaques of demyelination [3] and axonal damage. The latter might be consequent to demyelination, but can also occur independently of myelin destruction [4,5] eventually leading to axonal atrophy and impaired neuronal signal transmission [6,7]. The chronic disease is accompanied by a progressive loss of patient ability to repair damage and reacquire lost functions [8].

Current treatments depend upon patient’s classification and response to therapy [9], but all the available drugs are mainly aimed at controlling inflammation and modulating patients’ immune response [10]. While these strategies can slow down MS evolution, no drugs able to repair MS-induced demyelination are available yet [11].

Recently, it was demonstrated that the activation of the G protein-coupled receptor (GPCR) GPR17 controls the progression of oligodendrocyte precursor cells (OPCs) to mature oligodendrocytes *in vitro* [12–16]. However, while GPR17 is needed to start OPC differentiation, the receptor has to be timely downregulated at later differentiation stages to allow terminal maturation. Any failure in this physiological downregulation, resulting in aberrant ad prolonged GPR17 upregulation, blocks OPCs at immature stages and delays myelination. Indeed, abnormal up-regulation of GPR17 was reported in several CNS disorders such as brain ischemia, spinal cord and traumatic brain injury, as well as in a rodent model of Alzheimer’s disease [12,16–19]. Accordingly, GPR17 overexpression in late OPCs *in vitro* [16] and in transgenic mice results in loss of oligodendrocytes as well as myelination arrest [15]. Its localization on the extracellular membrane of myelinating cells and its behavior in pathophysiological conditions make GPR17 an attractive ‘druggable’ target [11,20–23]. Small molecules highly-selective for this receptor could be developed and used either alone or in synergy with other drugs able to control immune response and inflammation. Although previous studies have shown that GPR17 non-selective antagonists may be beneficial in limiting brain damage in acute conditions [12,13] we postulate that, in chronic diseases such as MS, GPR17 agonists may promote oligodendrocyte maturation and foster myelination, by promoting its signalling and the consequent receptor downregulation [24,25]. However, all the GPR17 agonists identified so far also interact with other receptors [26,27], which obviously hampers the development of these ligands into therapeutic agents [28].

The present study was purposely aimed at developing novel and highly selective GPR17 agonists *via* a rational drug discovery pipeline.

First, we modelled the three-dimensional structure of GPR17 [29,30] and afterwards carried out virtual high-throughput screening (HTS) for the identification of putative ligands [31,32], whose structures were then optimized *in silico* to ameliorate their affinity for GPR17. Among these novel selected ligands, some were tested in highly specific *in vitro* assays characterized by increasing complexity: i) a reference pharmacological assay for GPCR activation to confirm compounds ability to bind GPR17 and determine their intrinsic activity [12,33], followed by ii) testing in an OPC-dorsal root ganglia (DRG) neuron co-culture myelination assay.

Subsequently, the doses and ability to reach the brain of the most promising hits were investigated *in vivo*, guided by *in silico* pharmacokinetics studies. Then, we determined target selectivity *in vitro* and validated the ability of the best emerging candidate to modify disease evolution *in vivo* in experimental autoimmune encephalomyelitis (EAE) [34], a well-established mouse model that presents clinical and pathological similarities to human multiple sclerosis. The findings reported here confirm the strength of our drug-discovery pipeline and prompt new experiments in which selective GPR17 ligands can be administered alone or in combination with anti-inflammatory drugs to foster endogenous remyelination.

## Materials and methods

### Homology modelling

The three-dimensional structure of the human GPR17 receptor was built by homology modelling, using the 2.5 Å resolution X-ray structure of the human CXCR4 deposited in RCSB Protein Data Bank as template (PDB, code: 3ODU) [35], as previously described by Sensi and coauthors [29]. Briefly, the homology modelling procedure was performed using the MOE Homology Model program of the Molecular Operating Environment (Chemical Computing Group, Montreal, Canada) with standard settings, starting from a multiple sequence alignment of the primary structures of a subgroup of structurally related class-A GPCRs, selected according to the presence of a conserved pair of cysteines, putatively engaged in an extracellular disulfide bridge linking the N-terminus with the extracellular loop 3 (EL3), as previously described [36]. The multiple sequence alignment was performed using the TM-Coffee algorithm, a module of the T-Coffee alignment package optimized for transmembrane proteins [37]. For the homology modelling procedure, the Amber12:EHT force field with the reaction field electrostatics treatment was used.

### *In silico* HTS

A virtual HTS of a large chemical library of approximatively 130,000 lead-like compounds was performed on GPR17 model using the Dock program contained in the MOE Simulation module. The initial chemical database was supplied by Asinex (Asinex Platinum Collection, http://www.asinex.com/), and corresponds to a lead-like structural library of commercial 2D compounds, providing diverse and cost-effective coverage of drug-like chemical space. Most of the included compounds are characterized by a high degree of drug-likeness, in accordance with Lipinski’s rule of 5. The GPR17 binding site was identified through the MOE Site Finder module.

Docking calculations were performed following a previously described workflow implemented in the MOE software [31]. Accordingly, two progressive docking cycles based on two different prioritization stages (placement and refinement) were applied, each one characterized by increasing levels of deepness to account for the decreasing number of compounds to be screened at each stage. In all these stages, GPR17 was treated as rigid receptor, while ligands conformations were freely sampled. All the docking procedures were performed using the MMFF94x force field. Solvation effects were calculated using the reaction field functional form for the electrostatic energy term. For the estimation of the binding free energy of the generated complexes two different scoring functions with different degrees of accuracy were used (see below). Before starting with the first stage (placement), 20,000 conformations were generated for each ligand by sampling their rotatable bonds. Triangle Matcher methodology was selected for generating the docking poses for the initial scoring. Within this methodology, poses are generated by aligning ligand triplets of atoms on triplets of alpha spheres in a systematic way. Duplicate complexes were removed, and the accepted poses, set to 1,000 for each ligand, were scored according to the London dG empirical scoring function [38].

For each ligand, the top scoring complex coming from the first docking stage was submitted to the second more accurate step (refinement) based on molecular mechanics (MM). Energy minimization was carried out using a conventional molecular mechanics setup, under MMFF94x force field. In order to speed up the calculation, all receptor atoms were held fixed, and residues over a 6 Å cut-off distance away from the pre-refined pose were ignored, both during the refinement and in the final energy evaluation. During the refinement, solvation effects were calculated using the reaction field functional form for the electrostatic energy term and a dielectric constant of 4. The final binding free energy was evaluated using the force-field based GBVI/WSA ΔG empirical scoring function [32].

All the ligands contained in the Platinum library were screened according to the above procedure; then, the 15 top scoring compounds were resubmitted to the same docking procedure, keeping for each one of them 300 poses. The 6 ligands associated with the lowest binding free energy scores (most favourable poses) were selected for a patentability study aimed at generalizing their chemical formulas.

Then, this database was populated with additional compounds, selected through a fingerprint-based similarity search performed with MOE, useful for generalizing the chemical structures into three families. Standard Tanimoto coefficient was used to compute chemical distances between molecules.

### Combinatorial library enumeration

Three different enumerative combinatorial libraries were subsequently generated from the generic chemical formula of Family I, through the MedChem Transformations tool of the MOE suite.

For generating the first database (DBI), the scaffold was functionalized by applying iteratively a set of transformation rules to specific attachment points (ports). The MedChem Transformation was carried out using default parameters by applying an interaction cut-off value of 50 and a Transformation Limit of 300,000 molecules. Minimization was carried out using the MMFF94x force field and the R-field method as solvation model. For the R-substituent belonging to the aryl ring, 3 different ports were defined for the ortho, meta and para position, respectively, setting a cut-off of 300,000 molecules for each arene substitution pattern. The whole database was filtered using the MOE SD Tool, in order to select, for the subsequent HTS, only non-reactive molecules satisfying Oprea's test for lead-likeness [39].

For generating the second library (DBII), the 2-[[3-(2-methoxyphenyl)-1H-1,2,4-triazol-5-yl]thio]-acetamide scaffold was enumerated by adding a set of 53 aromatic heterocycles designed *ad hoc* (DBII) as R-groups through the MOE MedChem Transformation module using default parameters and setting 10 as Interaction limit.

The third database (DBIII) was generated by adding a dataset of commercially available aromatic amines as R-group to the 2-[[3-(2-methoxyphenyl)-1H-1,2,4-triazol-5-yl]thio]-acetamide scaffold, through the specific MOE “Add Group to Ligand” tool. The input database was obtained from the Aldrich Market Select chemical database (www.AldrichMarketSelect.com). Briefly, before enumeration, the library (originally containing 6784 entries) was pre-processed with the Database preparation tool for filtering and washing operations; then, each aminic nitrogen atom of the resulting library (6769 entries) was transformed in a hydrogen leaving group, and specific attachment points were defined using the MOE clip R-group function.

All the three distinct datasets were submitted to a multistage molecular docking workflow. In addition, in this case, after the first virtual HTS step, in which the binding free energy for each ligand::receptor complex was computed through the London dG scoring function, and only one solution for each ligand was kept, all the complexes were rescored and ranked according to their GBVI/WSA dG binding free energy score. After rescoring, the best 1,000 top-scoring molecules for each database were submitted to a more extensive docking procedure consisting of a placement stage, followed by a refinement step based on an explicit MM force field method.

During the placement and refinement stages, poses were scored according to the London dG and to the GBVI/WSA dg scoring functions, respectively, and only the best 30 poses were kept for each step. The same docking procedure was applied also to a chemical library of 5162 commercially available compounds (DBIV) from the Aldrich Market Select chemical database.

Finally, the 30 top-scoring complexes emerged from either DBI, DBII and DBIII, were optimized by applying the LigX procedure, in which MM minimization of both the ligand and the receptor binding site are performed. A detailed explanation of the computational strategy is reported also in Supplementary Material.

### [^35^S] GTPγS binding assay

[^35^S]GTPγS binding assays were carried out as previously described [25,31]. Briefly, control and astrocytoma 1321N1 cells stably transfected with human pcDNA3.1 (control cells) and human HA-tag GPR17 were homogenized in 5 mM Tris/HCl and 2 mM EDTA (pH 7.4) and centrifuged at 48,000g for 15 min at 4°C. The resulting pellets were washed in 50 mM Tris/HCl and 10 mM MgCl_2_ (pH 7.4). After protein dosage, aliquots of cell membranes were incubated with increasing concentrations of each investigated ligand (1 pM–10 μM) and GTPγS binding to activated G-proteins was quantified as previously described. Compounds were purchased from two external providers, i.e., Ambinter (Ambinter c/o Greenpharma, Orléans, France http://www.ambinter.com/) and Asinex (http://www.asinex.com/).

For analysis and graphic presentation of [^35^S]GTPγS binding data, a nonlinear multipurpose curve fitting computer program (Graph-Pad Prism) was used. All data are presented as the mean ± SEM of three different experiments.

### *In silico* ADME prediction

The ADME profile of the investigated compounds was predicted through the Schrödinger QikProp tool (Small-Molecule Drug Discovery Suite 2015–1, Schrödinger, LLC, New York, NY), which uses an algorithm based on the correlation between experimentally determined properties and Monte Carlo statistical mechanics simulations of organic solutes in a periodic box of explicit water molecules.

In parallel, ADME was predicted also through a combination of quantitative structure-activity relationship (QSAR) models, as implemented in the ACD/Percepta ADME Suite predictions (ACD/Labs, Toronto, Canada).

### *In vitro* myelination assay

OPC-DRG co-cultures were prepared according to a previously described protocol [14,40].

DRG explants from E14.5 mice were directly put in culture after being plucked off from embryo spinal cords. Then, they were grown in Neurobasal medium (Life Technologies) in presence of 100 ng/ml nerve growth factor (NGF, Sigma-Aldrich), and in presence of 10 μM fluorodeoxyuridine (FUDR, Sigma-Aldrich) in order to remove all non-neuronal cells [41].

Purified primary rat OPCs were obtained from mixed glial cultures by orbital shaking for 3 hours. The cell suspension was then centrifuged at 1200 rpm for 7 minutes, the supernatant was carefully discarded and the pellet was resuspended and dissociated in 6.5 ml of NM15 containing MEM (Life Technologies), 15% foetal bovine serum (FBS, Euroclone), 2 mM L-Glutamine (Euroclone), 6 mg/ml Glucose (Sigma-Aldrich), 5 μg/ml Insulin (Sigma-Aldrich), 100 U/ml Penicillin-100 μg/ml Streptomycin (Euroclone). The cell suspension was incubated for 20 minutes at room temperature with one of Ran-2 coated-dishes, which were previously rinsed 3 times with sterile PBS. Non-attached cells were collected and re-incubated for further 20 minutes with a second Ran-2 coated-dish. After incubation, the cell suspension was centrifuged at 1200 rpm for 10 minutes. Finally, the supernatant was discarded, and the pellet was resuspended in MEM, 10% FBS, 4 g/L Glucose, 2 mM L-Glutamine 100 U/ml Penicillin-100 μg/ml Streptomycin.

After 20 days, when neurites were well extended radially from DRG explants, purified OPCs were seeded onto DRG (35,000 cells/DRG) and kept in medium consisting of MEM, 10%FBS, 2mM L-Glutamine and P/S. In the so-obtained co-cultures, myelination was induced by adding 1 μg/ml TrkA-Fc (Space Import-Export). Tested compounds were added to cultures at day 4 at a final concentration of 10 nM, in parallel to vehicle as control (CTRL). The pharmacological treatment was repeated every 2 days up to day 15, when the cells were fixed for immunocytochemical analysis.

### Immunocytochemistry

At day 15, OPC/DRG co-cultures treated with either the selected compounds or the vehicle were fixed and treated with rat anti-MBP (Millipore), mouse anti-SMI 31 and mouse anti-SMI 32 (Cell Signaling), followed by the secondary goat anti-rat/-mouse antibody, conjugated to Alexa Fluor 555 or Alexa Fluor 488 (Life Technologies). The quantification of myelin segments was performed following a previously described protocol [42] with ZEISS LSM Image Browser by acquiring 6 random fields of 4–5 coverslips for each experimental condition. Stacks of images of MBP and Smi31/Smi32 positive cells were taken at 40X magnification; images in the stack were merged at each level and pixels overlapping in the red and green fields above a predefined threshold intensity value were highlighted in white. The amount of myelin per axon (myelination index) was calculated as the ratio between the white pixels area and the green pixels area.

To account for pseudo-replication, a linear mixed effects model (lmer procedure of lme4 R package) was used for statistical analysis:

Myelination index~Treatment+(1|Coverslip)+(1|Experiment)

where Treatment was considered as fixed effect and coverslip and experiment as random effects.

### *In vivo* mouse DMPK study

*In vivo* DMPK studies were performed as an external service by Aptuit (Verona, Italy) according to standard procedures.

Two compounds were tested in a single dose pharmacokinetic (PK) study in mouse, in which a 1 mg/kg single dose solution (15% Ethanol, 85% PEG400) was administered by subcutaneous injection to naïve CD1 male mice from accredited supplier (n = 3), weighed prior to dosing. Blood samples were collected for 8 time-points (0.25, 0.50, 1, 2, 4, 6, 8, 24 hours).

Plasma samples were prepared and diluted according standard in-house procedures and stored at approximately -20°C, pending analysis. Briefly, blood samples were collected into tubes containing anticoagulant (K_3_EDTA), stored on ice and centrifuged to prepare plasma within 2 hours of collection. Plasma samples were diluted in Hepes Buffer and deproteinised using CH_3_CN containing rolipram as internal reference compound for positive ion mode, according standard procedure (20 ng/ml). After vortexing and centrifugation (3000 rpm per 10 min), sample supernatants were transferred in 96 well plate and diluted with water for analysis.

Blood concentrations of the tested compounds and their metabolites were detected through Liquid Chromatography-tandem Mass Spectrometry (QTRAP® 4000 LC-MS/MS System, Sciex). The results for each test compound in plasma were subjected to non-compartmental PK analysis using WinNonlin Phoenix v.6.3 for generation of appropriate PK parameters, reported along with graphical representation of the mean plasma concentration profiles over time.

At 24 hours, animals were sacrificed, and brains were collected and processed for measuring compound concentrations through mass spectrometry.

Afterwards, the presence of the top compound in the brain was assessed in a single dose time course study consisting of a subcutaneous administration of a 10 mg/kg dose (15% Ethanol, 85% PEG400) to 24 naïve C57BL/6 male mice. Eight time-points (n = 3/time-point) were drawn over 24 hours after dosing. Each animal was sampled for blood collection at two time-points (the first via a tail vein, the second via cardiac puncture under anaesthesia). Blood samples were collected and centrifuged to prepare plasma according to standard procedures and stored at approximately -20°C, pending analysis (n = 48 samples). The brain of each animal was collected at sacrifice and analysed alongside plasma samples for compound quantification (n = 24 samples). Plasma and brain homogenate samples were analysed for test compound quantification using an optimised method based on protein precipitation followed by HPLC-MS/MS analysis. The results for test substance in plasma and brain homogenates were subjected to non-compartmental PK analysis using WinNonlin Phoenix v.6.3 for generation of appropriate pharmacokinetic parameters to be reported along with graphical representation of the mean plasma concentration profiles over time.

### Selectivity profile

The selectivity of the top compound versus a subset of selected class-A GPCRs was assessed through either binding or functional assays performed by Eurofins Cerep (Le Bois l'Evêque, France), as an external service. For evaluating both agonist and antagonist effects, a 100 nM solution of the top compound was tested against the 40 human receptors, listed in S2 Table, using known ligands as reference. Depending upon the assay, the ability of the top compound to target a specific GPCR was detected through scintillation counting, cellular dielectric spectroscopy, and fluorimetry.

### Animal health and experimental design

The Università degli Studi di Milano–La Statale (Italy) is compliant with all applicable national (D.Lgs. 26/2014) and European (Directive 2010/63/EU) regulations, for using animals in scientific research. All the experiments were approved by the Animal Care Committee of the Università degli Studi di Milano–La Statale, which is legally entitled for the use of animals for scientific purposes and by the Italian Ministry of Health (Authorization #473/2012-PR, 05/06/2015).

Twenty-four 6-week-old female C57BL/6N mice (Mus musculus; Charles River Laboratories Italia, Milan, Italy) were obtained and acclimatized for 2 weeks. Vendor health reports indicated that the animals were free of known viral, bacterial and parasitic pathogens. Animals were housed for a maximum of 3 per cage under a 12-h light/12-h dark cycle at 21C, with food and water *ad libitum* and in the presence of environmental enrichment. Before carrying out any procedure, the animals were subjected to a 2-week acclimatization period. Animal health was monitored daily to assess the presence of any signs of suffering. In particular, the parameters that were considered to assess the severity of the suffering and to define the endpoints beyond which the animal would be immediately suppressed, after consultation with the designated veterinary doctor, were: 1) lack of spontaneous intake of food and water for a 24 hour period; 2) rapid loss of body weight (up to 20% of the initial weight); 3) persistent hypothermia; 4) weakness; 5) visible and persistent changes in the coat (shine, hair loss, etc.); 6) reduced movement of the animal in the cage for at least 48h; 7) changes in grooming behaviour (for at least 48h); 8) blood loss from the orifices; 9) accelerated breathing for at least 24h; 10) incontinence or continuous diarrhoea for at least 48 hours. After surgery mice were placed over a heating mat to counteract hypothermia. Eyewash was used to counteract dry eyes. The experiments were designed in compliance with the ARRIVE guidelines. Control groups were included in all experiments, randomizing the procedures and applying blinded analysis when possible. Sample size was calculated with G-Power, to achieve a significant difference of P 0.05 and a power of 0.8.

### EAE induction and drug delivery

A 10 μg/μl solution of the top compound in 15% ethanol and 85% PEG (vehicle) was freshly prepared and loaded into 28-day Alzet mini-osmotic pumps (infusion rate of 0.11 μl/hour for 28 days) with a reservoir volume of 100 μl (Alzet, Charles River), so that each animal received 1.5 mg/Kg/die. This concentration had been calculated based on DMPK studies to reach a blood levels compatible with *in vitro* [^35^S]GTPγS EC_50_ values. Mini-pumps were incubated in saline at 37°C overnight to ensure a steady pump rate.

One day before immunization, mice were divided in two groups (n = 12 per group) based on their weight, in order to have similar mean weight between groups (control group = 19.5 ± 1.1 g; treated group = 19.1 ± 1.4 g). Then, the two groups received, in blind, subcutaneous implantation on the back of the mini-pumps, loaded with either vehicle or compound 9. The following day, EAE induction was performed as previously described [43]. Briefly, EAE was induced by subcutaneous immunization, with 200 μg of myelin oligodendrocyte glycoprotein (MOG_35-55_) per mouse. Immunized mice received 500 ng of pertussis toxin (PTX) intravenously the day of immunization and 48 h later. For both immunization and mini-pump implantation, mice were anesthetized with isoflurane.

Clinical scores were assessed daily, in blind, according to the following scale: 0 = healthy, 1 = flaccid tail, 2 = ataxia and/or paresis of hindlimbs, 3 = paralysis of hindlimbs and/or paresis of forelimbs, 4 = tetraparalysis, 5 = moribund or dead. For each animal, the onset day was recorded as the day post-immunization (dpi), corresponding to the appearance of the first clinical manifestations (score>0). The specific human points (HEP) indicated in the severity assessment framework for the EAE model were evaluated. In particular, we monitored mantle conditions, breathing, bladder control, nest conditions and social interactions. To avoid suffering related to food deprivation, at the first signs of weakness or paralysis of the limbs, fluid food was introduced into the cage. In addition, the amount of litter was increased to allow the animals to reach the water dispenser with minimum difficulty, and daily consumption was monitored. In this study it was not necessary to practice euthanasia.

Animals were anesthetized with ketamine (100 mg/kg) / xylazine (10 mg/kg) and sacrificed at day 28 after treatment.

The clinical outcomes analysed were: i) weight and clinical score changes during disease course between the two groups and ii) change in the day of onset between the two groups. Data are presented as mean ± SEM of DPI and were analysed with the GraphPad Prism 7.04 software. Shapiro-Wilk normality test was performed to assess normal distribution of data. One sample two tailed t-test was performed to assess data statistical significance. P < 0.05 was considered as statistically significant.

Five mice did not show any symptom along the whole experiments; 2 mice rejected the minipump few days after implantation. Thus, 7 animals were excluded from analysis (3 control and 4 treated mice).

## Results

### *In silico* identification of new hit compounds for GPR17

An iterative step-by-step procedure, consisting of HTS and combinatorial chemistry, was carried out to identify new potential hit agonists able to operate GPR17.

#### High-throughput screening

**Fig 1:**
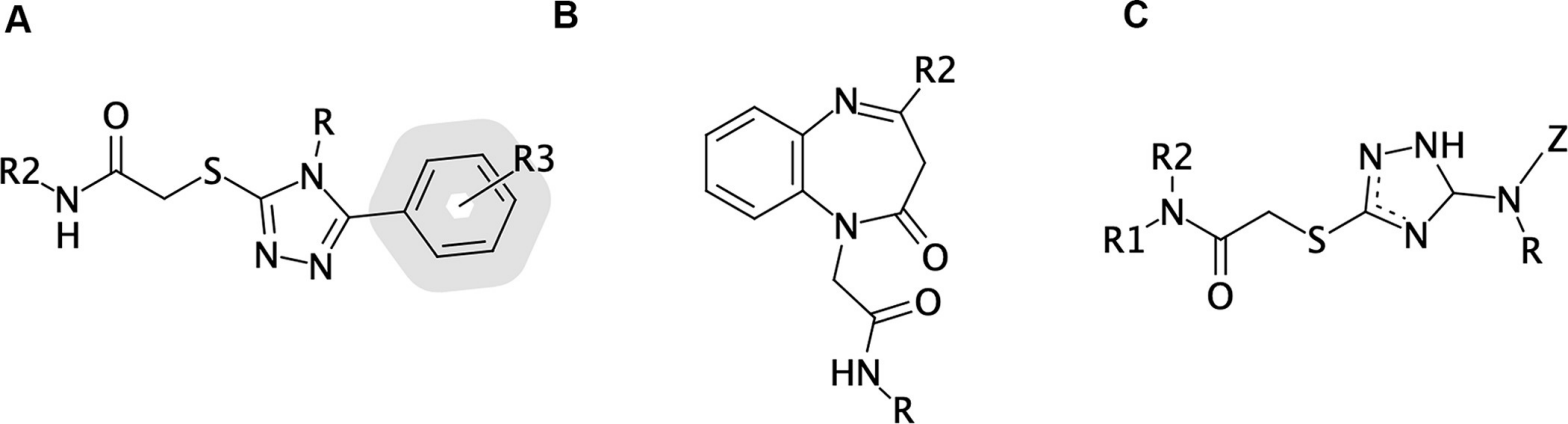
Generic chemical formula of the 3 patented families. (A) Family I; (B) Family II, (C) Family III.

HTS of a large database of commercially available lead-like compounds provided by Asinex was performed on the GPR17 three-dimensional model, driving the molecular docking calculations into the binding site identified through MOE, encompassing the residues previously identified as crucial for the recognition of orthosteric GPR17 ligands [29,31,44]. The docking solutions were ranked according to their predicted binding free energy values. The 6 top-scoring molecules coming from this first step (S1 Fig, compounds 1–6) were found to belong to three distinct structural clusters. These compounds were subjected to patentability evaluation aimed at generalizing their chemical structures. In parallel, a similarity search within the database of the 1,000 top-scoring docking solutions was also performed to make the general chemical formulas of the hit structures as inclusive as possible and to extend them to a chemical progeny already included in the database. As a result, the three identified families were populated with a total of 29 compounds, now protected by a Patent Cooperation Treaty (PCT/EP2012/058500). The three chemical families derived from these structures are reported in Fig 1.

#### Combinatorial expansion of Family I

Among the 3 chemical families, priority was given to Family 1, which, in a patent prior art search, resulted the most promising in view of a future deployment. The expansion of its general structure (Fig 1A) was performed through *in silico* combinatorial chemistry. To enhance the diversity of the chemical library, an iterative procedure was applied, which included chemical expansion of the scaffold, evaluation of results, and *in silico* screening, coupled to analysis of the drug-like properties and filtering according to drug-likeness [39].

Thanks to this strategy, a large combinatorial library containing diverse molecule subsets (each one produced with a different enumerative approach) was prepared, generating a final database of more than 1,000,000 compounds, as described in detail in Supplementary Material. This procedure allowed us to identify, among the whole set of explored R-groups in position R, R^2^, and R^3^ of the scaffold, specific requirements that increase the *in silico* affinity for GPR17, such as a hydrogen atom, an aromatic amine group and an ortho-methoxy-aryl group for R, R^2^ and R^3^, respectively.

**Fig 2:**
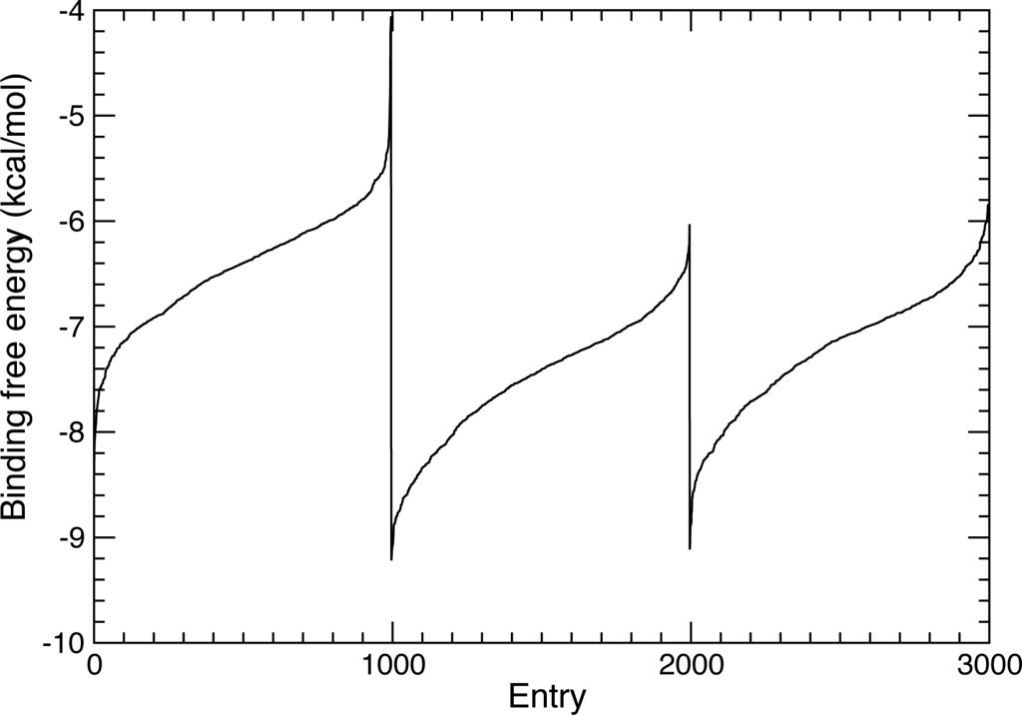
Binding free energy plots of the 1,000 top-scoring compounds selected for each combinatorial subset after complete docking procedure. Binding free energies, computed through the force field based GBVI/WSA ΔG empirical scoring function and sorted according to ascending order, are shown in progressive order for DBI, DBII, DBIII, respectively.

As shown in Fig 2, comparison of the evolution of binding free energies computed for the 1,000 top-scoring compounds of each diverse chemical subset shows that the introduced chemical modifications, such as the cyclization of–R^2^ with aromatic groups, progressively led to an improvement of the affinity for GPR17 of the designed compounds.

In order to speed-up the drug-discovery pipeline, the final database was checked for similarity across a library of commercial chemicals characterized by either a *N-*phenyl-2-[(3-phenyl-1*H*-1,2,4-triazol-5-yl)thio]- or a 2-[(4,5-diphenyl-4*H*-1,2,4-triazol-3-yl)thio]-*N*-phenyl-‘aromatic’ amide scaffold.

Binding free energy values obtained for the two different subgroups of compounds (R = H, phenyl ring) are reported in S2 Fig. For all the compounds in which–R was replaced by a hydrogen atom the binding free energy values were lower than those obtained for the compounds holding a phenyl ring, confirming that the complete removal of this group does not affect their ability to recognize GPR17 but instead increases affinity.

**Fig 3:**
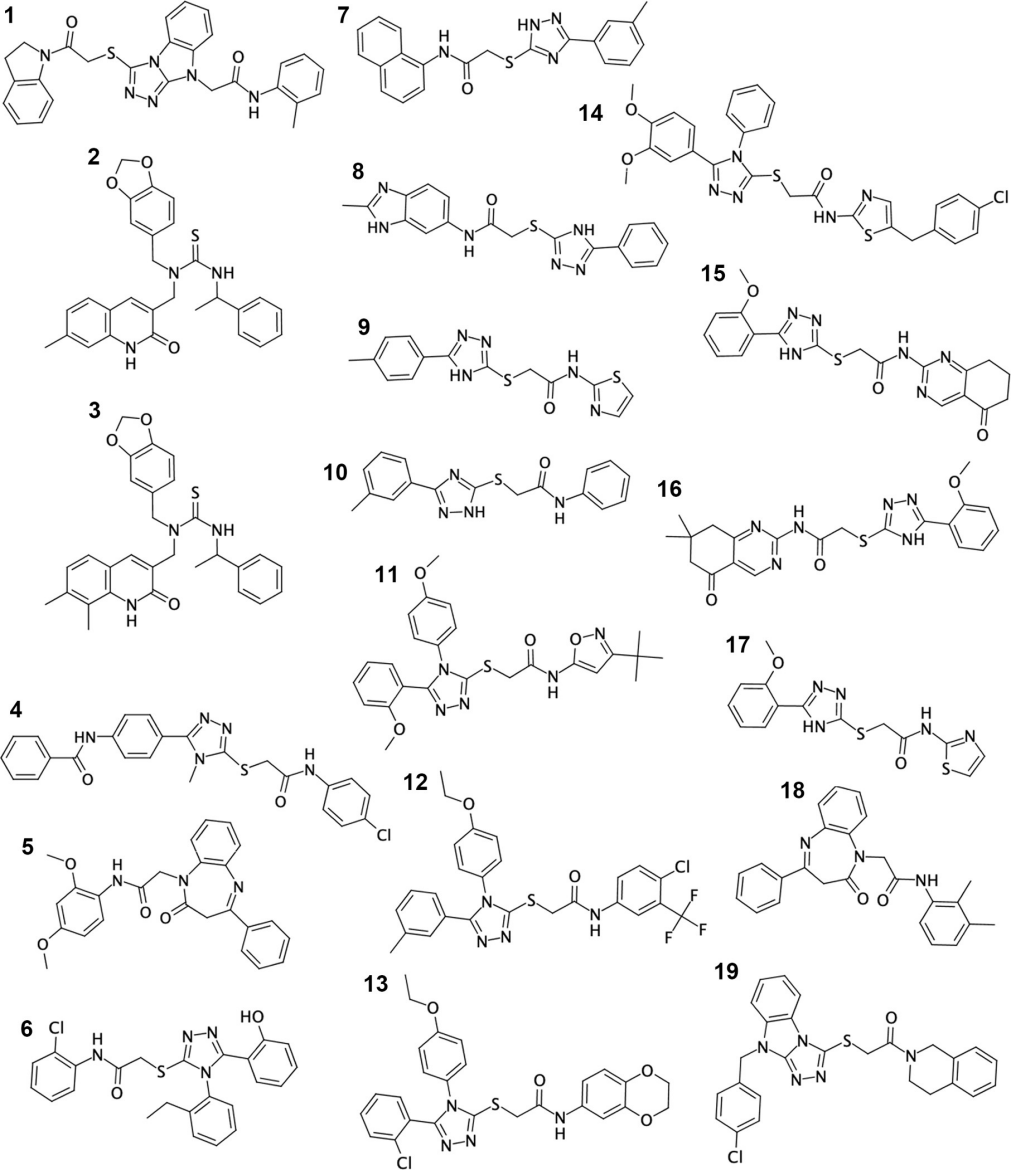
Chemical structures of the hit compounds selected through *in silico* HTS on GPR17. Compounds from 1 to 6 are the progenitors of the three chemical families (see also S1 Fig) identified through the earliest virtual screening. Together with compounds 4 and 6, compounds from 7 to 19 were selected among the ~1,000,000-compound library for preliminary *in vitro* testing. Compounds from 7 to 17 belong to Family I; compound 18 and 19 are representative of Family II and III, respectively.

Then, the combinatorial chemistry databases were searched against commercially available compounds. Thanks to this analysis, some of the top-scoring compounds from HTS turned out to be commercially available. In order to select the most promising hits to be further investigated and to validate our discovery strategy, a collection of 15 chemically diverse molecules, chosen among all the hit compounds derived from the whole *in silico* pipeline, was selected for *in vitro* testing. The structures of these compounds are reported in Fig 3. Indeed, the first 6 compounds reported in Fig 3 (compounds 1–6), are the progenitors of the three chemical families (see also S1). Compounds tested through the [^35^S]GTPγS are indexed with the following numbers: 4, 6, 7, 8, 9, 10, 11, 12, 13, 14, 15, 16, 17, 18, 19. Thirteen out of the 15 tested compounds, namely numbers 4, 6, 7–17, belong to Family I. Thus, to enhance the heterogeneity of the chemical structures to be tested, and to collect preliminary data on at least one lead compound *per* chemical family, as a back-up strategy, also members of both Family II (18) and Family III (19) were included in this analysis. While 18 has indeed a completely different structure from that of Family I, 19 resembles a cyclized form of the Family I scaffold.

A representative docking pose of one of the 15 selected compounds at the GPR17 binding site is shown in S3 Fig.

### *In vitro* [^35^S]GTPγS binding activity

The selected 15 molecules were evaluated in our in-house [^35^S]GTPγS functional assay in membranes from cells transfected with human GPR17. Human 1321N1 cells were selected as this cell line does not constitutively express GPR17. Moreover, this assay has been already successfully used in the past to measure the ability of both endogenous and novel synthetic compounds to activate GPR17 [25,31]. All tested molecules were able to potently activate GPR17 with nanomolar/sub-nanomolar affinities. Affinity and efficacy values compared to basal or to LTD_4_, here used as a reference agonist [12,13], are reported in Table 1. Concentration-response curves obtained for each selected compound are reported in S4 Fig.

**Table 1:** [^35^S]GTPγS binding assay.

Compound	EC_50_ (nM)	Emax (% *vs* basal)	% Emax (ratio *vs* Emax of the standard)
^**†**^**4**	0.43 ± 0.08	160.0 ± 10.6	110.8
^**†**^**6**	0.52 ± 0.09	135.0 ± 10.2	93.5*
^**†**^**7**	0.48 ± 0.09	151.3 ± 6.7	104.8
^**†**^**8**	0.24 ± 0.02	144.8 ± 6.4	100.3
^**†**^**9**	0.64 ± 0.12	127.8 ± 3.0	88.5**
^**†**^**10**	0.24 ± 0.06	142.0 ± 5.8	98.3
^**¶**^**11**	22.5 ± 4.30	134.3 ± 8.8	95.3
^**¶**^**12**	7.11 ± 1.96	127.7 ± 4.1	90.6*
^**¶**^**13**	3.29 ± 0.28	141.3 ± 7.6	100.2
^**¶**^**14**	2.70 ± 0.40	139.3 ± 2.5	98.8
^**¶**^**15**	2.90 ± 0.10	137.1 ± 1.3	97.23
^**¶**^**16**	3.45 ± 0.50	139.6 ± 1.5	99.0
^**¶**^**17**	8.48 ± 0.90	136.2 ± 0.5	96.6
^**¶**^**18**	1.68 ± 0.10	147.5 ± 5.8	104.6
^**¶**^**19**	9.50 ± 1.47	153.7 ± 1.1	108.5*
^**†**^LTD_4_ 50 nM		144.4 ± 7.1	100.0
^**¶**^LTC_4_ 50 nM		141.0 ± 3.9	100.0

* P< 0.05.

** P< 0.01 vs LTD_4_ or LTC_4_ set to 100%.

### *In silico* ADME prediction

The absorption, distribution, metabolism, and excretion (ADME) properties of the 15 tested compounds, in comparison with the same properties for well-characterized drugs, were simulated *in silico*, using two different approaches. Data from the two analyses were then crosschecked to select the best hits for further *in vitro* and *in vivo* testing. Among the selection criteria, we gave priority to predicted CNS penetration and chemical stability. Also, physicochemical descriptors and other general properties connected with overall good DMPK profiles were considered, such as good oral and intestinal absorption, plasmatic transport, etc.

Among all the selected compounds, 9 showed the best *in silico* DMPK profile, on the basis of a set of more than 100 descriptors and/or ADME models, as described in the Materials and methods section. Among all the investigated properties, brain penetration was considered as a mandatory feature for the selected compounds. The most relevant DMPK properties computed for compound 9 are recapitulated in Table 2; essential DMPK parameters for progenitors of Family II and Family III are reported alongside for comparison. In view of its promising properties, compound 9 soon attracted our attention, including receiving a fantasy name - galinex.

**Table 2:** Most relevant *in silico* DMPK parameters for the selected compounds.

	Recommended range	Compound 9	Compound 18	Compound 19
LogP		2.68	3.62	5.72
Number of violations of Lipinski’s rule of five	<4	0	2	1
Number of violations of Jorgensen’s rule of three	<3	0	2	1
Number of property or descriptor value outside the 95% range of similar values for known drugs	0–5	0	2	2
Predicted brain/blood partition coefficient	-3.0 –+1–2	-0.958	-0.407	-0.197
^1^MDCK cell permeability in nm/sec	<25 poor	536	1029	4503
	>500 great			
Number of reactive functional groups	0–2	0	1	0
Number of likely metabolic reactions	1–8	3	7	4
^2^Predicted apparent Caco-2 cell permeability in nm/sec	<25 poor	446.138	1969.945	1506.359
	>500 great			
Prediction of the binding to human serum albumin	-1.5 –+1.5	-0.041	0.0672	0.905
Prediction of human oral absorption	<25% poor	88.5	100	100
	>80% high			
Brain penetration sufficient for CNS activity		yes	yes	low
Absorption across intestinal barrier		yes	yes	yes
Passive absorption across intestinal barrier		good	good	good
First-pass metabolism in liver and/or intestine		no	no	yes

^1^MDCK cells are a well-established model for BBB.

^2^Caco-2 cells are a well-established model for the gut-blood barrier.

Previous *in vitro* assay showed that all the tested compounds are potent GPR17 activators, and therefore they all result of potential interest from a pharmacological point of view. *In silico* DMPK data were then used to prioritize molecules to be further investigated. Together with compound 9, as a backup strategy, also compound 18, which belongs to a different chemical class, was progressed to the subsequent *in vivo* DMPK phase.

### Determination of *in vivo* pharmacokinetic properties

ADME parameters for both 9 and 18 were evaluated in an *in vivo* single dose DMPK study in mice. Essential DMPK values for the two molecules, after a single subcutaneous administration of a 1 mg/kg dose, are reported in Table 3. Plasma concentrations used for deriving these data are reported in S1 Table.

**Table 3:** *In vivo* DMPK plasma parameters of compounds 9 and 18.

Plasma DMPK parameters	9	18
Cmax (ng/ml)	72.3	26.4
Tmax (h)	0.50	1.00
Clast (ng/ml)	4.81	1.00
Tlast (h)	4.00	4.00
AUClast (ng h/ml)	85.8	46.7
t1/2 (h)	1.09	0.759

Compound 9 was characterized by more favourable PK parameters than 18, suggesting a higher bioavailability and a better *in vivo* manageability.

The metabolic stability of the two compounds was also analysed. DMPK experiments showed that 9 is poorly metabolized; in fact, no detectable metabolites were found *in vivo* under the selected experimental conditions. In contrast, 18 generated 5 major metabolites, whose plasmatic concentrations are reported in S5 Fig. These data are in good agreement with the *in silico* predictions suggesting chemical stability for 9, but not for 18.

Overall, 9 showed a better DMPK profile in comparison with 18, and was thus selected for further evaluations.

To verify whether 9 was indeed able to reach the CNS, the presence of this compound in mouse brain after subcutaneous administration of a single dose of 10 mg/kg was assessed in a time-course experiment.

The following DMPK parameters were found for 9 in mice brain, when dosed at 10 mg/kg: C_max_ = 270 ng/ml, T_max_ = 0.25 h; Clast 5.80 ng/ml, T_last_ = 6.00 h, AUC_last_ = 205 h*ng/ml. In parallel, the plasma concentration of this compound was evaluated, generating the following PK parameters: 10 mg/kg: C_max_ = 6520 ng/ml, T_max_ = 0.25 h; Clast 17.4 ng/ml, T_last_ = 8.00 h, AUC_last_ = 3930 h*ng/ml.

In line with activity at picomolar concentrations, as obtained *in vitro* (EC_50_ = 0.64 ± 0.12 nM), these data confirm our hypothesis that 9 may display activity in the CNS, as predicted by our *in silico* approach.

### *In vitro* pro-myelinating activity

**Fig 4:**
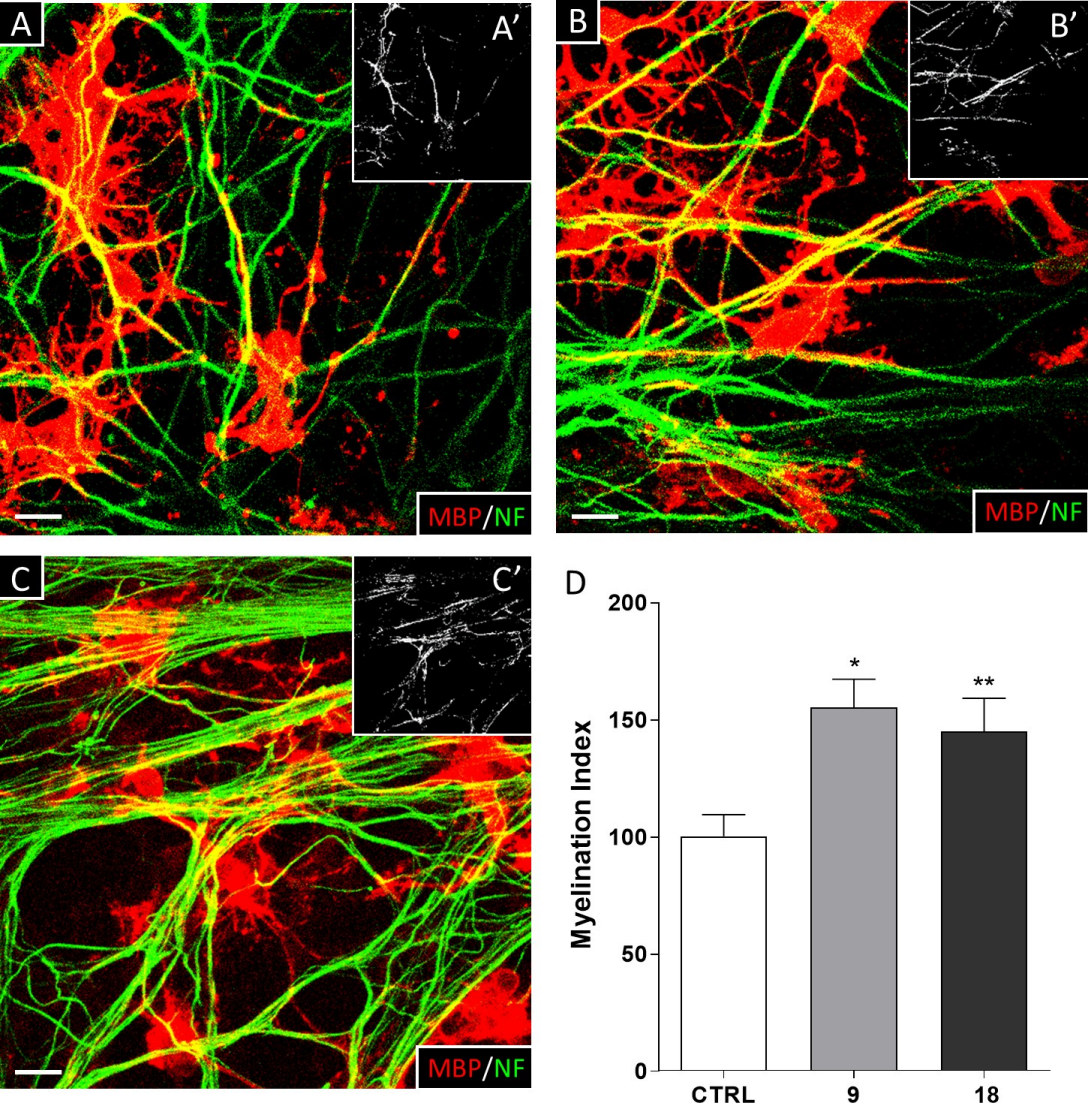
Effect of compounds 9 and 18 on myelin deposition in OPC-DRG co-cultures. Representative micrographs of OPC/DRG co-cultures treated with either vehicle (A), or compound 9 (B) or compound 18 (C). Immunostaining for anti-MBP antibody and anti-neurofilament (NF) antibodies are shown in red and green fluorescence, respectively. The myelination rate is represented by the colocalization of MBP with NF (in yellow) and is highlighted in the insets as white pixels (A’, B’, C’); scale bar = 20μm. (D) Histograms show the quantification of myelin segments (Myelination Index). Data are expressed as mean ± S.E.M. of the Myelination Index obtained from the analysis of 5 random fields of 5 coverslips for each experimental condition from 3 independent experiments. One-way ANOVA with Tukey’s multiple comparisons test, * p < 0.05, ** p < 0.01 vs vehicle group.

On the basis of *in silico* and *in vivo* DMPK data, which showed a promising pharmacokinetic profile for 9, we decided to further progress to *in vitro* validation. In parallel, also 18 was tested as a backup strategy. We thus tested the ability of these compounds to promote myelination in the in-house *in vitro* model of myelination on primary OPC-DRG neuronal co-cultures [40,41,45]. As shown in Fig 4, both compounds were able to significantly promote the formation of newly myelinated segments positive for the Myelin Basic Protein (MBP^+^) when tested at a concentration of 10 nM, as demonstrated by the increased value of myelin segments (Myelination Index, CTRL: 100 ± 9.28%; compound 9: 155.0 ± 12.08%, and compound 18: 144.7 ± 14.28%). This increase was associated to the presence of more MBP^+^-myelinated axons in the compound-treated cultures compared to vehicle-treated ones. Based on the *in vitro* myelinating data, we decided to further progress compound 9 to the subsequent *in vivo* study.

### Selectivity profile

Before proceeding to the *in vivo* study, the affinity of compound 9 for a total of 40 structurally and phylogenetically related GPCRs, including purinergic P2Yn, CysLT1 and CysLT2 and chemokine receptor sub-families (for selection of investigated receptors, please, see [36]) was evaluated through classical binding assays, when available, or through cellular assays. A subset of GPCRs with a relevant role in demyelination/remyelination was also included in this analysis.

We found that, at the tested concentration of 0.1 μM, 9 exhibits no significant pharmacological activity on all the tested GPCRs. Results from this screening are collected in S2 and S3 Tables, showing data from binding and cellular assays, respectively.

Since, for the selectivity studies, 9 was tested at a concentration markedly higher than its *in vivo* active concentration (approx. 156-fold over its EC_50_ value), no off-target effects are expected for this compound, at least for the investigated GPCRs.

These results suggest that compound 9 is extremely selective for GPR17 and a promising candidate for *in vivo* studies.

### Effect of compound 9 (galinex) in the EAE mouse model

Then, to evaluate if compound 9 was able to act as a disease-modifying agent *in vivo*, we administered it to EAE mice according to a chronic preventive protocol. Compound 9 was loaded into osmotic mini pumps implanted subcutaneously on the back of mice to obtain a continuous 28-day infusion starting on the day before disease induction. The 1.5 mg/kg/die-dose was determined making reference to the *in silico* prediction and *in vivo* DMPK experiments described above.

**Fig 5:**
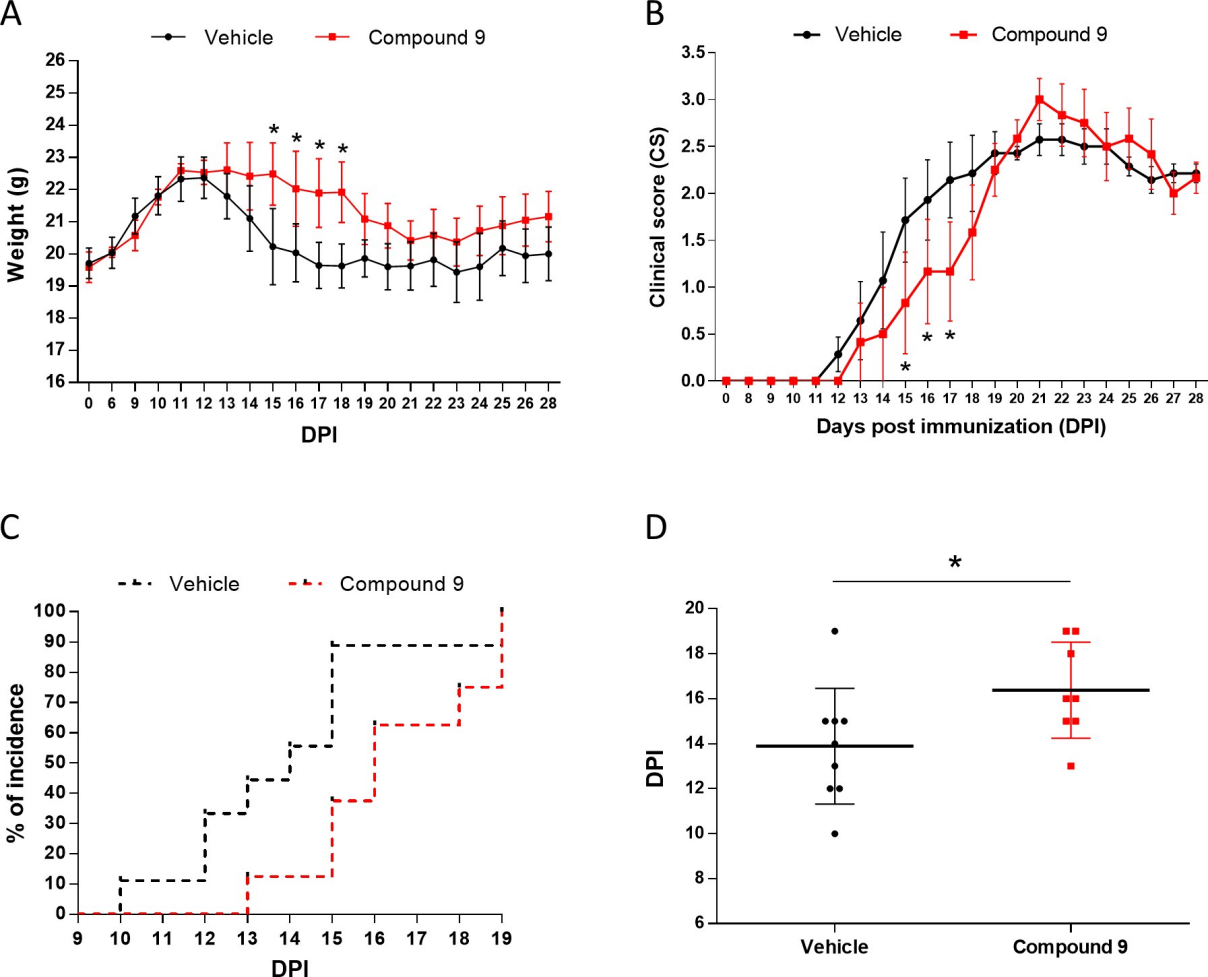
Evaluation of the activity of compound 9 (galinex) *in vivo* in the EAE mouse model. (A, B) Weight and clinical scores (CS) of EAE mice during disease course (vehicle-treated group in black, galinex-treated group in red). Error bars represent mean of CS/weight ± SEM. Multiple t test; * p<0.05. (C) Kaplan-Meier curves showing EAE incidence in vehicle- and galinex-treated mice. Incidence is reported as percentage of diseased mice/group size. Gehan-Breslow-Wilcoxon test; p-value = 0.034. (D) Day of EAE onset in vehicle (15% ethanol, 85% PEG; n = 9) and compound 9 (galinex; 10 μg/μl in vehicle; n = 8) treated mice. Data are expressed as the mean ± S.E.M of DPI. Unpaired two-tailed Student’s test, * p<0.05.

Both weight and clinical score were monitored daily during EAE. Compound 9 globally attenuated the weight loss typically observed during disease course, which was significant from 15 to 18 days after induction (Fig 5A). We also observed that compound 9 significantly reduced the clinical scores in the early phases of EAE compared to controls (Fig 5B). Both experimental groups reached a 100% incidence of disease at the end of the observation period, but the Kaplan-Meier curve of compound 9-treated mice was shifted compared to the control group (Fig 5C), suggesting a delaying effect on disease onset. Symptoms onset was indeed significantly postponed by 2.5 days (Fig 5D).

Globally, these results suggest that, by activating GPR17, compound 9 has a protective activity on EAE development, thus delaying the appearance of neurological symptoms. This represents the first evidence in favour of the *in vivo* disease-modifying properties of a selective GPR17 agonist.

## Discussion

Here, we propose and validate an iterative drug discovery pipeline through which novel putative GPR17 modulators have been designed and then validated using screening paradigms proceeding from *in silico* simulations to an *in vivo* model of disease. At each run, this pipeline is characterized by increasing degrees of complexity and an increasingly stringent selection of the compounds on the basis of their drug likeness. More than 1,000,000 compounds were filtered through this step-by-step production chain, ending with one single molecule that indeed proved to be effective *in vivo*, thus validating the predictivity of the whole procedure (S6 Fig). Accordingly, our results demonstrate that the proposed strategy could largely supersede serendipitous or exclusively wet-based approaches [46,47].

As a first step, we needed to develop an *in silico* model of our target. We did so using class-A GPCR comparative modelling, a well-known bioinformatics strategy aimed at gathering novel structural information on this receptor family that indeed encompasses about 70% of all drug-targets [48,49]. Although GPR17 experimental structure has not been solved yet, the availability of homologous proteins to be used as templates, combined with specific class-A GPCR modelling techniques [50,51], allowed us to obtain an accurate and validated model, with a well-shaped binding site, useful for the subsequent HTS procedures [29,31,36,52,53]. Accordingly, in the very last years, homology modelling has been successfully applied also by other groups for identifying both similar and chemically diverse GPR17 ligands [23,54].

High-performance molecular docking of large chemical libraries was then used to quickly and inexpensively prioritise chemicals according to their affinity for GPR17, and immediately deliver them to subsequent more in-depth tests, useful to select the most promising hits.

*In silico* combinatorial chemistry was used to efficiently explore drug-like chemical space of potential novel hits, and to increase chemical diversity. The use of commercial chemical regents for exploding the parent structures was used to overcome the synthetic bottleneck and make chemical synthesis easier. However, to quickly obtain a validation of our pipeline, our combinatorial chemistry databases were searched against commercially available compounds, characterised by a high degree of drug-likeness in accordance with specific organic chemistry rules, such as Lipinski’s [55] and Oprea’s [39] rules.

After these initial phases, use of a well-established *in vitro* reference pharmacological assay for class-A GPCRs, namely the GTPγS binding assay [33], allowed us to classify the chemical hits according to classical pharmacological parameters, such as potency, efficacy and intrinsic activity, and to validate our computational procedure.

All the *in silico* identified compounds resulted being GPR17 agonists, with nanomolar EC_50_ values.

As molecular docking estimates binding free energy, this approach is not intrinsically able to specifically pick up agonists from an *in silico* screening if a quantitative structure–activity relationship (QSAR) model is not available, as is the case for GPR17. However, these results are not surprising since all compounds of Family I derive from a common scaffold with agonist activity. Of note, previous independent virtual screening on the receptor led to the identification of GPR17 agonists with demonstrated promyelinating activity on *in vitro* rodent models characterized by similar structure [23,31,44] and potency, suggesting that this scaffold may be suitable for defining a lead compound for GPR17. Moreover, this potency is in line with that of other class-A GPCR agonists [56], and of our previous set of novel GPR17 activators [31].

*In silico* DMPK evaluations, through which compounds were ranked according to various parameters (including drug-likeness, metabolic stability, oral absorption, affinity to plasma proteins, ability to penetrate the BBB, etc), were useful to expedite the identification of compounds to be forwarded to *in vivo* DMPK experimental validation.

As a backup strategy, alongside with the most promising hit of Family I (i.e., compound 9, or galinex), also a reference compound from Family II (compound 18) was selected for further *in vitro* and *in vivo* evaluations. No further activities were pursued on chemicals from Family III, since the latter can be considered as a cyclized derivative of Family I.

As expected from the accumulated evidence on GPR17 role in oligodendrocyte differentiation [13,14,24], both these agonists promoted myelination in a well-established OPC-DRG co-culture model [14,40].

Such promising findings, together with the good *in silico* and *in vivo* DMPK properties of compound 9, led us to eventually test it in EAE mice, a model resembling several clinical features of human MS and the most credited model for testing the efficacy of disease modifying compounds [34]. Based on DMPK studies, that revealed relatively short half-life, a constant infusion through Alzet osmotic minipumps was set-up as administration route for the EAE model, in order to guarantee accurate blood levels of compound 9 for the whole treatment time, as well as to avoid to animals stressful conditions due to repeated administrations. The preventive administration of compound 9 significantly delayed the symptomatic onset of EAE, suggesting the predictivity of our *in silico* and *in vitro* pipeline, which has therefore the potential to be successfully used to identify other *in vivo* protective compounds. Based not only on specific *in silico* strategy, but also on the selectivity study carried out on the 40 most similar and more phylogenetically closely related class-A GPCRs [44], we believe the beneficial effects of 9 be due to a specific protective interaction with GPR17 on resident oligodendrocytes, the only cells expressing the receptor in this model [15,20]. However, since one of the main challenges for new chemical entities are the off-target effects, future studies should be also focussed on the investigation of other possible non-specific targets of this compound. Moreover, further analysis in the pre-symptomatic phase will be necessary to confirm this hypothesis and also to evaluate a possible anti-inflammatory/antioxidant/immunomodulatory effect of 9.

We have previously demonstrated that prolonged activation of GPR17 leads to receptor desensitization and internalization [24]. Thus, we cannot exclude that the beneficial *in vivo* effect of the GPR17 agonist presented here could be a consequence of receptor adaptive changes due to long term agonist administration *in vivo*. This could explain why, in some *in vitro* studies, GPR17 inactivation at specific oligodendrocyte maturation stages could also improve oligodendrocyte differentiation [21]. In a similar way, in the middle cerebral artery occlusion model, acute treatment with the non-selective antagonist cangrelor resulted in a reduction of the brain infarct size [13]. Finally, in old mice showing typical signs of cerebral degeneration, chronic treatment with another non-selective antagonist (Montelukast) was proved to fully revert the associated cognitive impairment through mechanisms specifically involving GPR17 [57]. Thus, differences in the administration protocol and in *in vivo* receptor adaptation events may account for the detected protective effects of both agonists and antagonists.

In the present paper, due to the severity of the experimental model, we have chosen a preventive administration protocol, reasoning that the continuous stimulation of GPR17 on resident intact OPCs during disease development could have been more effective in contrasting MOG-induced myelin deterioration, rather than the intervention on already established severe myelin damage with a curative protocol. Thus, the beneficial effects shown by compound 9 in the preventive protocol are likely due to a myelin protective activity, rather that indicative of remyelination. Future studies using a therapeutic protocol in the chronic phase of EAE will be necessary to test the actual in vivo remyelinating capability of compound 9.

However, since we cannot exclude that the strong pro-inflammatory environment associated to later EAE stages may inhibit the activity of compound 9, a combination treatment with an anti-inflammatory drug might be necessary. As this compound proceeds towards the preclinical development, future efforts should also take into account the optimization of a formulation and a route of administration associated to higher compliance, such as an oral administration.

## Conclusions

The present data confirm the effectiveness of our drug discovery pipeline in the identification of novel and selective GPR17 modulators, and show, for the first time, that a selective GPR17 agonist can effectively alter disease development *in vivo*. It is currently believed that a strategy in which myelin protection is combined with immune suppression and/or inflammation alleviation to face all MS components at once could be more effective and ameliorate disease prognosis [58]. Thus, we believe that targeting GPR17 with new selective compounds in combination with the already available immunosuppressive/anti-inflammatory drugs could represent a promising reparative strategy for MS and other neurodegenerative diseases characterized by demyelination.

## Supporting information

S1 Text *In silico* combinatorial expansion of Family I. (PDF)

S1 Fig Chemical structures of the 6 top-scoring compounds selected through *in silico* HTS on GPR17. Binding free energy values for the selected compounds, computed according to the force field based GBVI/WSA ΔG empirical scoring function, are: -35.60 kcal/mol for 1 (A); for 2 (B); -33.02 kcal/mol for 3 (C); -32.57 kcal/mol for 4 (D); -32.20 kcal/mol for 5 (E); -31.98 kcal/mol for 6 (F).
(TIF)

S2 Fig Binding free energy plots of the top-scoring compounds from DBIV. Binding free energies, computed through the force field based GBVI/WSA ΔG empirical scoring function and sorted according to ascending order, are shown as black and red lines for the 2-[(4,5-diphenyl-4*H*-1,2,4-triazol-3-yl)thio]-*N*-phenyl- (in black) and the *N-*phenyl-2-[(3-phenyl-1*H*-1,2,4-triazol-5-yl)thio]- (in red) ‘aromatic’ amide scaffold, respectively.
(TIF)

S3 Fig Docking pose of compound 9 on GPR17 binding site. The GPR17 3D model is shown in cartoon representation and coloured according to MOE GPCR annotation. Compound 9 docked in GPR17 binding site is shown in stick representation. The ligand::receptor interaction surface computed as van der Waals accessible surface is shown as yellow shell.
(TIFF)

S4 Fig Pharmacological profile of selected top-scoring compounds on GPR17. [^35^S]GTPγS binding assay dose-response curves for compounds 4, 6, 7–19. The endogenous GPR17 ligand LTD_4_ was used as reference compound. All data are expressed as percentage of basal [^35^S]GTPγS binding (set to 100%) and are mean ± SEM of 3 different experiments, each one performed in duplicate.
(TIF)

S5 Fig *In vivo* metabolic profile of compound 18 after subcutaneous administration. Compound identified as 18 is reported as Parent; expected metabolites are designated with M followed by a progressive number.
(TIFF)

S6 Fig Drug discovery pipeline. (TIFF)

S1 Table Plasma concentrations of compounds 9 and 18 after subcutaneous administration in mice. (PDF)

S2 Table *In vitro* pharmacological binding assays of compound 9 on selected GPCRs. (PDF)

S3 Table *In vitro* pharmacological functional assays of compound 9 on selected GPCRs. (PDF)

## References

[1] Mayo L, Quintana FJ, Weiner HL. The innate immune system in demyelinating disease. Immunol Rev. 2012;248: 170–187. 10.1111/j.1600-065X.2012.01135.x 22725961 PMC3383669

[2] Leray E, Moreau T, Fromont A, Edan G. Epidemiology of multiple sclerosis. Rev Neurol (Paris). 2016;172: 3–13. 10.1016/j.neurol.2015.10.006 26718593

[3] Kutzelnigg A, Lucchinetti CF, Stadelmann C, Br??ck W, Rauschka H, Bergmann M, et al Cortical demyelination and diffuse white matter injury in multiple sclerosis. Brain. 2005;128: 2705–2712. 10.1093/brain/awh641 16230320

[4] Haines JD, Inglese M, Casaccia P. Axonal damage in multiple sclerosis. Mt Sinai J Med. 2011;78: 231–43. 10.1002/msj.20246 21425267 PMC3142952

[5] Trapp BD, Nave K-A. Multiple Sclerosis: An Immune or Neurodegenerative Disorder? Annu Rev Neurosci. 2008;31: 247–269. 10.1146/annurev.neuro.30.051606.094313 18558855

[6] Compston A, Coles A. Multiple sclerosis. The Lancet. 2008 pp. 1502–1517. 10.1016/S0140-6736(08)61620-7 18970977

[7] Dutta R, Trapp BD. Mechanisms of neuronal dysfunction and degeneration in multiple sclerosis. Progress in Neurobiology. 2011 pp. 1–12. 10.1016/j.pneurobio.2010.09.005 20946934 PMC3030928

[8] Fitzner D, Simons M. Chronic progressive multiple sclerosis - pathogenesis of neurodegeneration and therapeutic strategies. Curr Neuropharmacol. 2010;8: 305–15. 10.2174/157015910792246218 21358979 PMC3001222

[9] Hurwitz BJ. The diagnosis of multiple sclerosis and the clinical subtypes. Ann Indian Acad Neurol. 2009;12: 226–30. 10.4103/0972-2327.58276 20182569 PMC2824949

[10] Goldenberg MM. Multiple sclerosis review. P T. 2012;37: 175–84. 22605909 PMC3351877

[11] Stangel M, Kuhlmann T, Matthews PM, Kilpatrick TJ. Achievements and obstacles of remyelinating therapies in multiple sclerosis. Nat Rev Neurol. 2017;13: 742–754. 10.1038/nrneurol.2017.139 29146953

[12] Ciana P, Fumagalli M, Trincavelli MLML, Verderio C, Rosa P, Lecca D, et al The orphan receptor GPR17 identified as a new dual uracil nucleotides/cysteinyl-leukotrienes receptor. EMBO J. 2006;25: 4615–4627. 10.1038/sj.emboj.7601341 16990797 PMC1589991

[13] Lecca D, Trincavelli ML, Gelosa P, Sironi L, Ciana P, Fumagalli M, et al The recently identified P2Y-like receptor GPR17 is a sensor of brain damage and a new target for brain repair. PLoS One. 2008;3: e3579 10.1371/journal.pone.0003579 18974869 PMC2570486

[14] Fumagalli M, Daniele S, Lecca D, Lee PRPR, Parravicini C, Douglas Fields R, et al Phenotypic changes, signaling pathway, and functional correlates of GPR17-expressing neural precursor cells during oligodendrocyte differentiation. J Biol Chem. 2011;286: 10593–10604. 10.1074/jbc.M110.162867 21209081 PMC3060511

[15] Chen Y, Wu H, Wang S, Koito H, Li J, Ye F, et al The oligodendrocyte-specific G protein-coupled receptor GPR17 is a cell-intrinsic timer of myelination. Nat Neurosci. 2009;12: 1398–406. 10.1038/nn.2410 19838178 PMC2783566

[16] Boda E, Vigano F, Rosa P, Fumagalli M, Labat-Gest V, Tempia F, et al The GPR17 receptor in NG2 expressing cells: Focus on in vivocell maturation and participation in acute trauma and chronic damage. Glia. 2011;59: 1958–1973. 10.1002/glia.21237 21956849

[17] Alavi MS, Karimi G, Roohbakhsh A. The role of orphan G protein-coupled receptors in the pathophysiology of multiple sclerosis: A review. Life Sci. 2019;224: 33–40. 10.1016/j.lfs.2019.03.045 30904492

[18] Ceruti S, Villa G, Genovese T, Mazzon E, Longhi R, Rosa P, et al The P2Y-like receptor GPR17 as a sensor of damage and a new potential target in spinal cord injury. Brain. 2009;132: 2206–2218. 10.1093/brain/awp147 19528093

[19] Franke H, Parravicini C, Lecca D, Zanier ER, Heine C, Bremicker K, et al Changes of the GPR17 receptor, a new target for neurorepair, in neurons and glial cells in patients with traumatic brain injury. Purinergic Signal. 2013;9: 451–462. 10.1007/s11302-013-9366-3 23801362 PMC3757149

[20] Coppolino GT, Marangon D, Negri C, Menichetti G, Fumagalli M, Gelosa P, et al Differential local tissue permissiveness influences the final fate of GPR17-expressing oligodendrocyte precursors in two distinct models of demyelination. Glia. 2018;66: 1118–1130. 10.1002/glia.23305 29424466 PMC5900886

[21] Merten N, Fischer J, Simon K, Zhang L, Schröder R, Peters L, et al Repurposing HAMI3379 to Block GPR17 and Promote Rodent and Human Oligodendrocyte Differentiation. Cell Chem Biol. 2018 10.1016/j.chembiol.2018.03.012 29706593 PMC6685917

[22] Lu C, Dong L, Zhou H, Li Q, Huang G, Bai SJ, et al G-Protein-Coupled Receptor Gpr17 Regulates Oligodendrocyte Differentiation in Response to Lysolecithin-Induced Demyelination. Sci Rep. 2018;8: 4502 10.1038/s41598-018-22452-0 29540737 PMC5852120

[23] Saravanan KM, Palanivel S, Yli-Harja O, Kandhavelu M. Identification of novel GPR17-agonists by structural bioinformatics and signaling activation. Int J Biol Macromol. 2018;106: 901–907. 10.1016/j.ijbiomac.2017.08.088 28827203

[24] Fratangeli A, Parmigiani E, Fumagalli M, Lecca D, Benfante R, Passafaro M, et al The regulated expression, intracellular trafficking, and membrane recycling of the P2Y-like receptor GPR17 in Oli-neu oligodendroglial cells. J Biol Chem. 2013/01/05. 2013;288: 5241–5256. 10.1074/jbc.M112.404996 23288840 PMC3576128

[25] Daniele S, Trincavelli ML, Gabelloni P, Lecca D, Rosa P, Abbracchio MP, et al Agonist-Induced Desensitization/Resensitization of Human G Protein-Coupled Receptor 17: A Functional Cross-Talk between Purinergic and Cysteinyl-Leukotriene Ligands. J Pharmacol Exp Ther. 2011;338: 559–567. 10.1124/jpet.110.178715 21531793

[26] Fumagalli M, Lecca D, Abbracchio MP. CNS remyelination as a novel reparative approach to neurodegenerative diseases: The roles of purinergic signaling and the P2Y-like receptor GPR17. Neuropharmacology. 2016 pp. 82–93. 10.1016/j.neuropharm.2015.10.005 26453964

[27] Hennen S, Wang H, Peters L, Merten N, Simon K, Spinrath A, et al Decoding signaling and function of the orphan G protein-coupled receptor GPR17 with a small-molecule agonist. Sci Signal. 2013;6: ra93 10.1126/scisignal.2004350 24150254 PMC4114018

[28] Plemel JR, Liu W-Q, Yong VW. Remyelination therapies: a new direction and challenge in multiple sclerosis. Nat Rev Drug Discov. 2017 10.1038/nrd.2017.115 28685761

[29] Sensi C, Daniele S, Parravicini C, Zappelli E, Russo V, Trincavelli ML, et al Oxysterols act as promiscuous ligands of class-A GPCRs: In silico molecular modeling and in vitro validation. Cell Signal. 2014;26: 2614–2620. 10.1016/j.cellsig.2014.08.003 25152366

[30] Spyrakis F, Cavasotto CN. Open challenges in structure-based virtual screening: Receptor modeling, target flexibility consideration and active site water molecules description. Archives of Biochemistry and Biophysics. 2015 pp. 105–119. 10.1016/j.abb.2015.08.002 26271444

[31] Eberini I, Daniele S, Parravicini C, Sensi C, Trincavelli ML, Martini C, et al In silico identification of new ligands for GPR17: A promising therapeutic target for neurodegenerative diseases. J Comput Aided Mol Des. 2011/07/12. 2011;25: 743–752. 10.1007/s10822-011-9455-8 21744154

[32] Wojciechowski M, Lesyng B. Generalized Born model: Analysis, refinement, and applications to proteins. J Phys Chem B. 2004;108: 18368–18376. 10.1021/jp046748b

[33] Strange PG. Use of the GTPγS ([35S]GTPγS and Eu-GTPγS) binding assay for analysis of ligand potency and efficacy at G protein-coupled receptors. British Journal of Pharmacology. 2010 pp. 1238–1249. 10.1111/j.1476-5381.2010.00963.x 20662841 PMC3000650

[34] Kipp M, Nyamoya S, Hochstrasser T, Amor S. Multiple sclerosis animal models: a clinical and histopathological perspective. Brain Pathology. 2017 pp. 123–137. 10.1111/bpa.12454 27792289 PMC8029141

[35] Wu B, Chien EYT, Mol CD, Fenalti G, Liu W, Katritch V, et al Structures of the CXCR4 chemokine GPCR with small-molecule and cyclic peptide antagonists. Science. 2010;330: 1066–71. 10.1126/science.1194396 20929726 PMC3074590

[36] Parravicini C, Daniele S, Palazzolo L, Trincavelli ML, Martini C, Zaratin P, et al A promiscuous recognition mechanism between GPR17 and SDF-1: Molecular insights. Cell Signal. 2016;28: 631–642. 10.1016/j.cellsig.2016.03.001 26971834

[37] Chang J-M, Di Tommaso P, Taly J-F, Notredame C. Accurate multiple sequence alignment of transmembrane proteins with PSI-Coffee. BMC Bioinformatics. 2012;13: S1 10.1186/1471-2105-13-S4-S1 22536955 PMC3303701

[38] Naïm M, Bhat S, Rankin KN, Dennis S, Chowdhury SF, Siddiqi I, et al Solvated Interaction Energy (SIE) for scoring protein-ligand binding affinities. 1. Exploring the parameter space. J Chem Inf Model. 2007/01/24. 2007;47: 122–133. 10.1021/ci600406v 17238257

[39] Oprea TI. Property distribution of drug-related chemical databases. J Comput Aided Mol Des. 2000;14: 251–264. 10.1023/a:1008130001697 10756480

[40] Fumagalli M, Bonfanti E, Daniele S, Zappelli E, Lecca D, Martini C, et al The ubiquitin ligase Mdm2 controls oligodendrocyte maturation by intertwining mTOR with G protein-coupled receptor kinase 2 in the regulation of GPR17 receptor desensitization. Glia. 2015;63: 2327–2339. 10.1002/glia.22896 26228571

[41] Taveggia C, Thaker P, Petrylak A, Caporaso GL, Toews A, Falls DL, et al Type III neuregulin-1 promotes oligodendrocyte myelination. Glia. 2008;56: 284–293. 10.1002/glia.20612 18080294

[42] Zhang H, Jarjour AA, Boyd A, Williams A. Central nervous system remyelination in culture - - a tool for multiple sclerosis research. Exp Neurol. 2011;230: 138–48. 10.1016/j.expneurol.2011.04.009 21515259 PMC3117145

[43] Furlan R, Cuomo C, Martino G. Animal models of multiple sclerosis. Methods Mol Biol. 2009;549: 157–73. 10.1007/978-1-60327-931-4_11 19378202

[44] Capelli D, Parravicini C, Pochetti G, Montanari R, Temporini C, Rabuffetti M, et al Surface Plasmon Resonance as a tool for ligand binding investigation of engineered GPR17 receptor, a G protein coupled receptor involved in myelination. Front Chem. 2019 10.3389/fchem.2019.00910 31998697 PMC6966494

[45] Chan JR, Watkins TA, Cosgaya JM, Zhang C, Chen L, Reichardt LF, et al NGF controls axonal receptivity to myelination by Schwann cells or oligodendrocytes. Neuron. 2004;43: 183–191. 10.1016/j.neuron.2004.06.024 15260955 PMC2758239

[46] Hughes JP, Rees SS, Kalindjian SB, Philpott KL. Principles of early drug discovery. British Journal of Pharmacology. 2011 pp. 1239–1249. 10.1111/j.1476-5381.2010.01127.x 21091654 PMC3058157

[47] Wadood A, Ahmed N, Shah L, Ahmad A, H H, Shams S. In-silico drug design: An approach which revolutionarised the drug discovery process. open Access Drug Des Deliv. 2013;1: 1–4. 10.13172/2054-4057-1-1119

[48] Bermudez M, Wolber G. Structure versus function - The impact of computational methods on the discovery of specific GPCR-ligands. Bioorganic Med Chem. 2015;23: 3907–3912. 10.1016/j.bmc.2015.03.026 25828056

[49] Stockert JA, Devi LA. Advancements in therapeutically targeting orphan GPCRs. Front Pharmacol. 2015;6: 100 10.3389/fphar.2015.00100 26005419 PMC4424851

[50] Costanzi S, Skorski M, Deplano A, Habermehl B, Mendoza M, Wang K, et al Homology modeling of a Class A GPCR in the inactive conformation: A quantitative analysis of the correlation between model/template sequence identity and model accuracy. J Mol Graph Model. 2016;70: 140–152. 10.1016/j.jmgm.2016.10.004 27723562 PMC5138091

[51] Ciancetta A, Jacobson K. Structural Probing and Molecular Modeling of the A3 Adenosine Receptor: A Focus on Agonist Binding. Molecules. 2017;22: 449 10.3390/molecules22030449 28287473 PMC5471610

[52] Parravicini C, Ranghino G, Abbracchio MP, Fantucci P. GPR17: Molecular modeling and dynamics studies of the 3-D structure and purinergic ligand binding features in comparison with P2Y receptors. BMC Bioinformatics. 2008;9: 263 10.1186/1471-2105-9-263 18533035 PMC2443813

[53] Parravicini C, Abbracchio MP, Fantucci P, Ranghino G. Forced unbinding of GPR17 ligands from wild type and R255I mutant receptor models through a computational approach. BMC Struct Biol. 2010;10: 8 10.1186/1472-6807-10-8 20233425 PMC2850907

[54] Baqi Y, Pillaiyar T, Abdelrahman A, Kaufmann O, Alshaibani S, Rafehi M, et al 3-(2-Carboxyethyl)indole-2-carboxylic Acid Derivatives: Structural Requirements and Properties of Potent Agonists of the Orphan G Protein-Coupled Receptor GPR17. J Med Chem. 2018;61: 8136–8154. 10.1021/acs.jmedchem.7b01768 30048589

[55] Lipinski CA, Lombardo F, Dominy BW, Feeney PJ. Experimental and computational approaches to estimate solubility and permeability in drug discovery and development settings. Advanced Drug Delivery Reviews Mar, 2012 pp. 4–17. 10.1016/j.addr.2012.09.019 11259830

[56] Alexander SP, Davenport AP, Kelly E, Marrion N, Peters JA, Benson HE, et al The Concise Guide to PHARMACOLOGY 2015/16: G protein-coupled receptors. Br J Pharmacol. 2015;172: 5744–5869. 10.1111/bph.13348 26650439 PMC4718210

[57] Marschallinger J, Schäffner I, Klein B, Gelfert R, Rivera FJ, Illes S, et al Structural and functional rejuvenation of the aged brain by an approved anti-asthmatic drug. Nat Commun. 2015;6: 8466 10.1038/ncomms9466 26506265 PMC4639806

[58] Fox RJ, Thompson A, Baker D, Baneke P, Brown D, Browne P, et al Setting a research agenda for progressive multiple sclerosis: The International Collaborative on Progressive MS. Mult Scler J. 2012/08/25. 2012;18: 1534–1540. 10.1177/1352458512458169 22917690 PMC3573679

