# Supplementary figures and images for: Development of the first in vivo GPR17 ligand through an iterative drug discovery pipeline: A novel disease-modifying strategy for multiple sclerosis
Source: PLoS One. 2020 Apr 22;15(4):e0231483. doi: 10.1371/journal.pone.0231483 (PMC7176092; doi:10.1371/journal.pone.0231483)

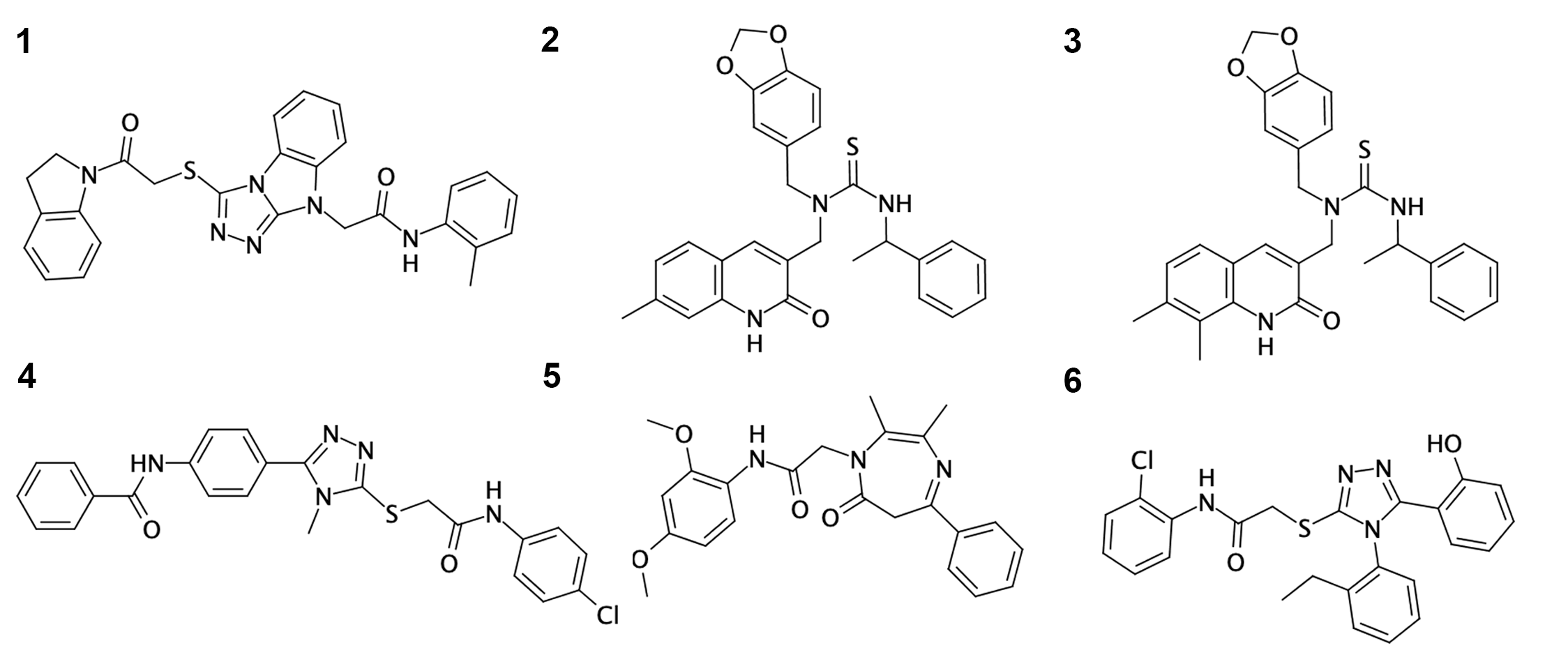

Supplement: S1 Fig — Binding free energy values for the selected compounds, computed according to the force field based GBVI/WSA ΔG empirical scoring function, are: -35.60 kcal/mol for 1 (A); for 2 (B); -33.02 kcal/mol for 3 (C); -32.57 kcal/mol for 4 (D); -32.20 kcal/mol for 5 (E); -31.98 kcal/mol for 6 (F). (TIF) [file pone.0231483.s002.tif]

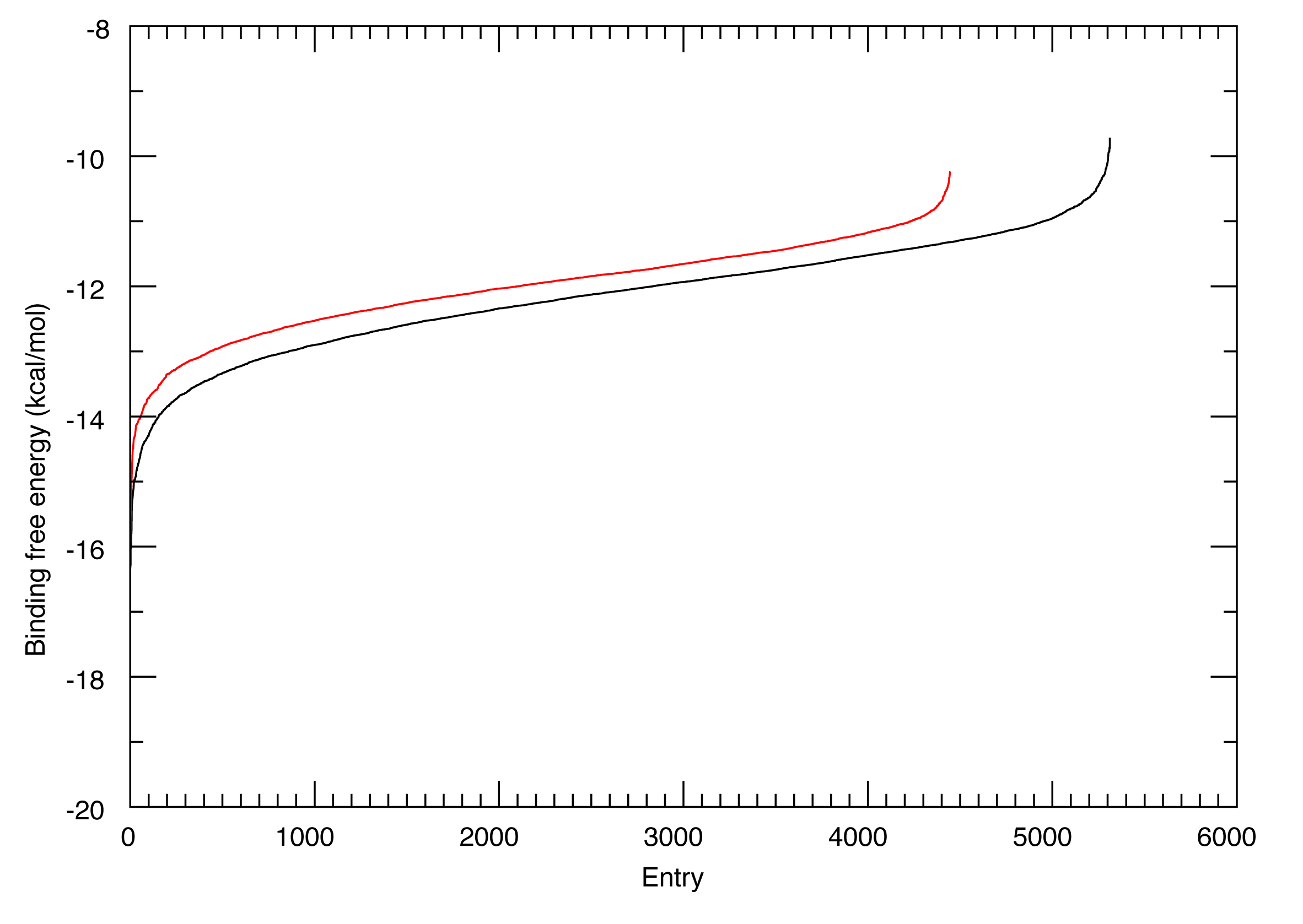

Supplement: S2 Fig — Binding free energies, computed through the force field based GBVI/WSA ΔG empirical scoring function and sorted according to ascending order, are shown as black and red lines for the 2-[(4,5-diphenyl-4H-1,2,4-triazol-3-yl)thio]-N-phenyl- (in black) and the N-phenyl-2-[(3-phenyl-1H-1,2,4-triazol-5-yl)thio]- (in red) ‘aromatic’ amide scaffold, respectively. (TIF) [file pone.0231483.s003.tif]

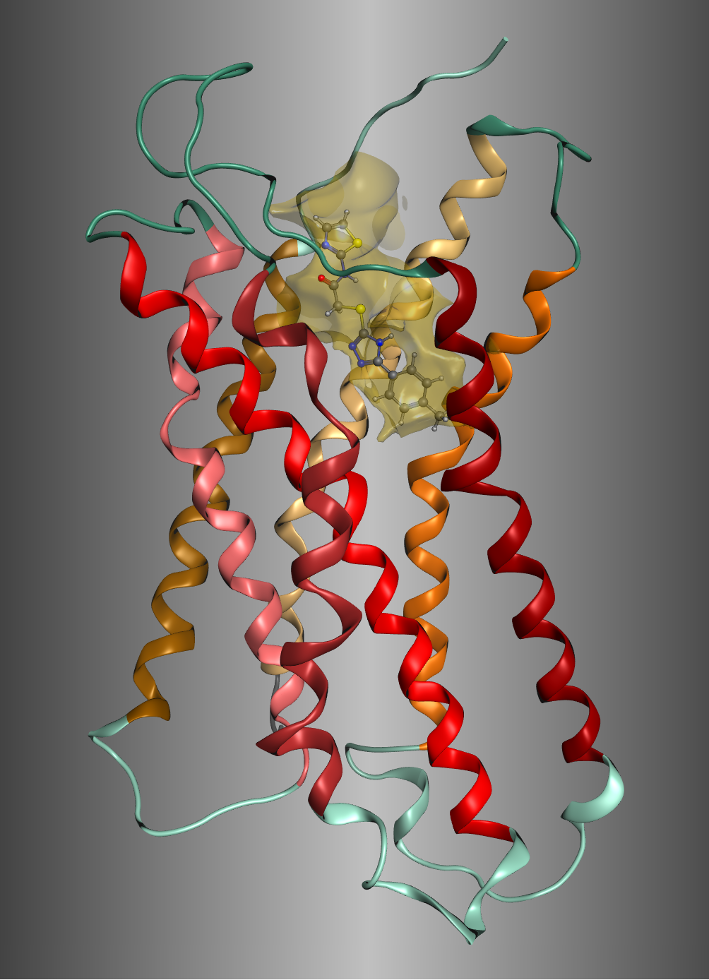

Supplement: S3 Fig — The GPR17 3D model is shown in cartoon representation and coloured according to MOE GPCR annotation. Compound 9 docked in GPR17 binding site is shown in stick representation. The ligand::receptor interaction surface computed as van der Waals accessible surface is shown as yellow shell. (TIFF) [file pone.0231483.s004.tiff]

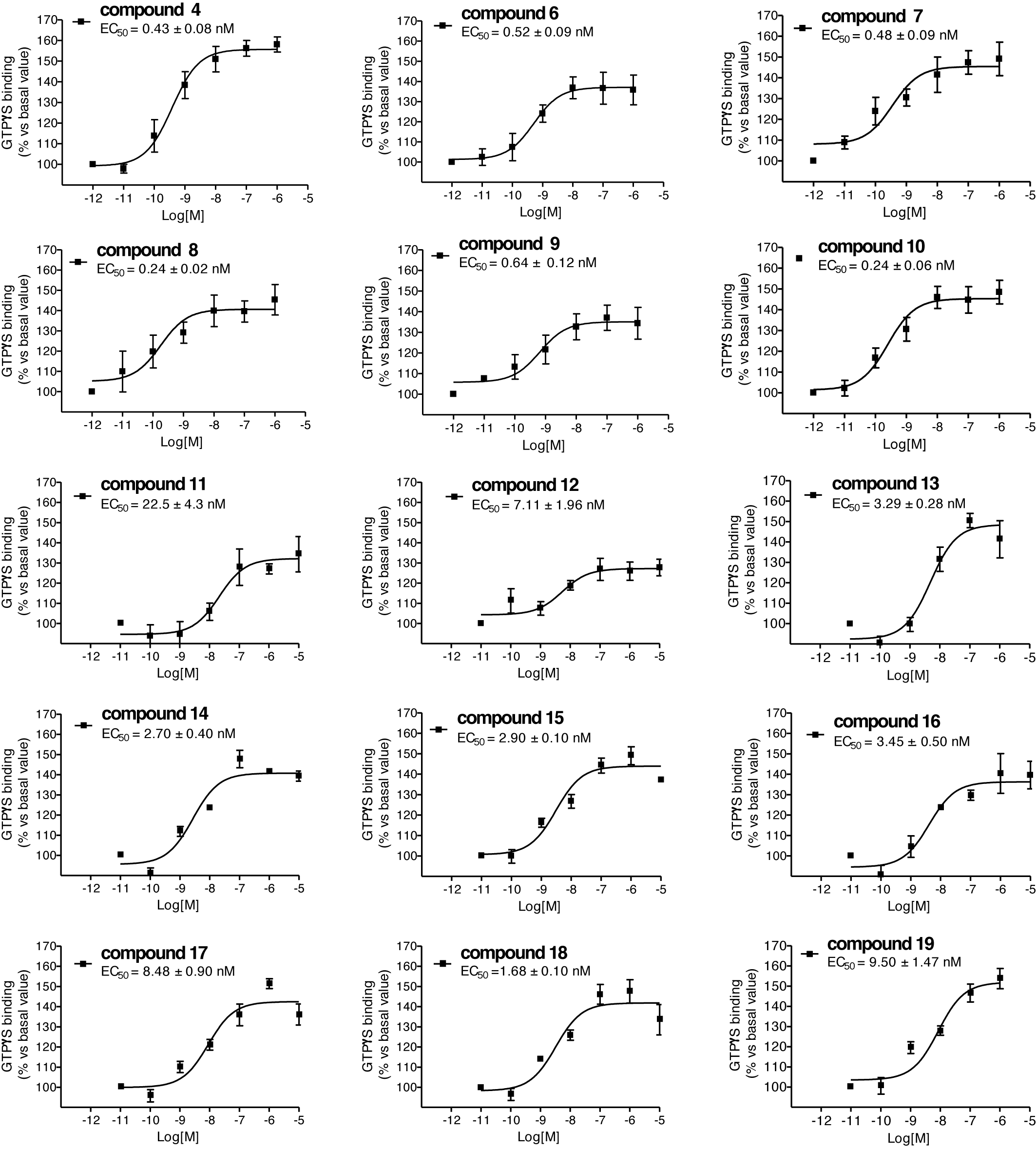

Supplement: S4 Fig — [35S]GTPγS binding assay dose-response curves for compounds 4, 6, 7–19. The endogenous GPR17 ligand LTD4 was used as reference compound. All data are expressed as percentage of basal [35S]GTPγS binding (set to 100%) and are mean ± SEM of 3 different experiments, each one performed in duplicate. (TIF) [file pone.0231483.s005.tif]

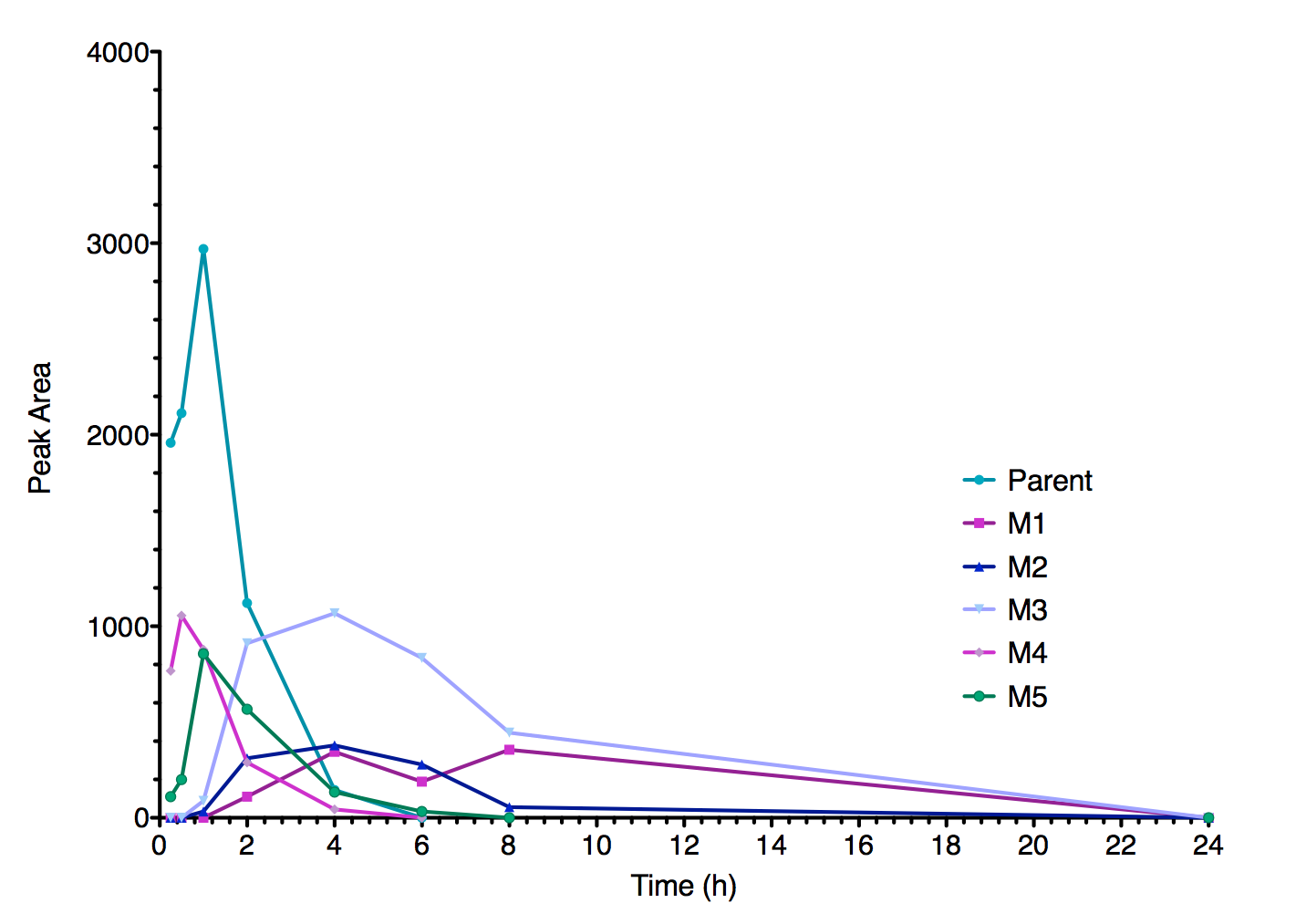

Supplement: S5 Fig — Compound identified as 18 is reported as Parent; expected metabolites are designated with M followed by a progressive number. (TIFF) [file pone.0231483.s006.tiff]

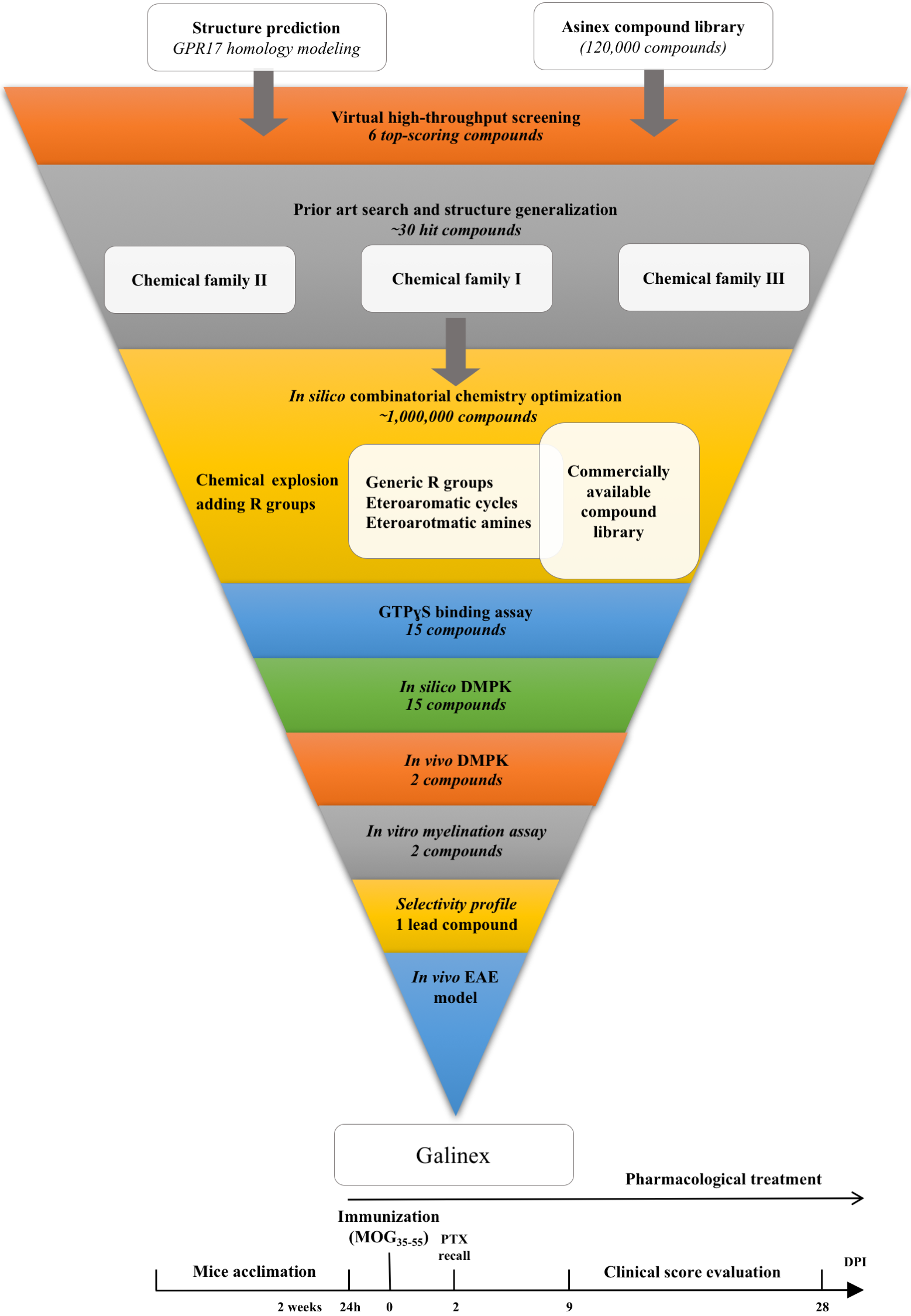

Supplement: S6 Fig — (TIFF) [file pone.0231483.s007.tiff]
