# Supplementary material for: Development of the first in vivo GPR17 ligand through an iterative drug discovery pipeline: A novel disease-modifying strategy for multiple sclerosis
Source: PLoS One. 2020 Apr 22;15(4):e0231483. doi: 10.1371/journal.pone.0231483 (PMC7176092; doi:10.1371/journal.pone.0231483)
Supplement: S1 Table — (PDF) [file pone.0231483.s008.pdf]

S1 Table. Plasma concentrations of compounds 9 and 18 after subcutaneous administration in mice

**Compound 9**					
**Time (h)**	**Mice n=1**	**Mice n=2**	**Mice n=3**	**Mean (ng/ml)**	**S.E.M.**
0.25	46.9	29.4	70	48.8	11.8
0.50	30.8	71.7	98.3	66.9	19.6
1.00	14.3	38.5	76.3	43.0	18.0
2.00	5.6	10.3	29.8	15.2	7.4
4.00	2.9	2.6	-	2.8	
6.00	-	-	-		
8.00	-	-	-		
24.00	-	-	-		
**Compound 18**					
**Time (h)**	**Mice n=4**	**Mice n=5**	**Mice n=6**	**Mean (ng/ml)**	**S.E.M.**
0.25	11.6	9.5	24.6	15.3	4.7
0.50	23.0	17.4	29.4	23.3	3.5
1.00	20.7	25.5	30.8	25.7	2.9
2.00	6.2	13.0	13.3	10.8	2.3
4.00	0.7	2.5	1.5	1.6	0.5
6.00	-	0.8	-		
8.00	-	-	-		
24.00	-	-	-		

Number of mice per compound = 3
