# Supplementary material for: Development of the first in vivo GPR17 ligand through an iterative drug discovery pipeline: A novel disease-modifying strategy for multiple sclerosis
Source: PLoS One. 2020 Apr 22;15(4):e0231483. doi: 10.1371/journal.pone.0231483 (PMC7176092; doi:10.1371/journal.pone.0231483)
Supplement: S2 Table — (PDF) [file pone.0231483.s009.pdf]

S2 Table. *In vitro* pharmacological binding assays of compound 9 on selected GPCRs

Assay	% Inhibition of control specific binding	% Of control specific binding		Mean	S.E.M.	IC_{50}	K_i
		1	2				
ADRα1A	12	116.7	108.1	112.4	4.3	0.39 nM	0.19 nM
ADRα1B	10	100.1	79.4	89.8	10.35	0.15 nM	40 pM
ADRα1D	-1	90.2	111.5	100.9	10.65	0.7 nM	0.3 nM
ADRα2A	0	100.3	99.5	99.9	0.40	6.3 nM	2.8 nM
ADRα2B	0	104.3	95.6	99.9	4.35	6.4 nM	4.3 nM
ADRα2C	-9	109.4	108.4	108.9	0.50	3.9 nM	1.3 nM
ADRβ1	9	94	88.9	91.5	2.55	0.33 μM	0.19 μM
ADRβ2	-2	95.8	108.1	101.9	6.15	1.1 nM	0.37 nM
ADRβ3	1	102.5	95.7	99.1	3.40	0.18 μM	0.14 μM
AT1	-10	112.1	108.6	110.4	1.75	0.79 nM	0.4 nM
APJ	-7	107.8	105.7	106.7	1.05	0.25 nM	0.22 nM
mGluR1	-14	103.5	124.2	113.8	10.35	0.16 μM	0.14 uM
mGluR5	-4	102.2	106.8	104.5	2.30	67 nM	14 nM
CXCR2	3	96	98.7	97.3	1.35	0.26 nM	0.12 nM
CCR1	-8	107.8	109	108.4	0.60	20 pM	13 pM
CCR2	12	88	88.9	88.4	0.45	72 pM	29 pM
CysLT1	2	89.7	105.4	97.6	7.85	0.68 nM	0.3 nM
CysLT2	-1	97.9	103.4	100.6	2.75	3.3 nM	2 nM
M1	-8	105.4	110.2	107.8	2.40	29 nM	25 nM
M2	-8	104.1	111.1	107.6	3.50	43 nM	30 nM
M3	-6	101.1	110.4	105.7	4.65	0.94 nM	0.67 nM
M4	5	95.7	95.1	95.4	0.30	1.2 nM	0.74 nM
M5	3	89.7	105.1	97.4	7.70	0.73 nM	0.37 nM
P2Y	-28	120.7	135.7	128.2	7.50	51 nM	25 nM

Compound binding was calculated as a % inhibition of the binding of a radioactively labeled ligand, either agonist or antagonist, specific for each target. Results showing an inhibition or stimulation higher than 50% are considered to represent significant effects of the test compounds. Number of replicates: 2. Reference compounds: ADRα1A: WB 4101; ADRα1B: prazosin; ADRα1D: prazosin; ADRα2A: yohimbine; ADRα2B: yohimbine; ADRα2C: yohimbine; ADRβ1: atenolol; ADRβ2: ICI 118551; ADRβ3: Alprenolol; AT1: saralasin; APJ: apelin-13:TFA; mGluR1: L-Quisqualate; mGluR5: L-Quisqualate; CXCR2: IL-8; CCR1: MIP-1α; CCR2: MCP-1; CysLT1: LTD_4; CysLT2: LTC_4; M1: pirenzepine; M2: methoctramine; M3: 4-DAMP; M4: 4-DAMP; M5: 4-DAMP; P2Y: dATPαS.
