# Supplementary material for: Development of the first in vivo GPR17 ligand through an iterative drug discovery pipeline: A novel disease-modifying strategy for multiple sclerosis
Source: PLoS One. 2020 Apr 22;15(4):e0231483. doi: 10.1371/journal.pone.0231483 (PMC7176092; doi:10.1371/journal.pone.0231483)
Supplement: S3 Table — (PDF) [file pone.0231483.s010.pdf]

S3 Table. ***In vitro*** **pharmacological functional assays of compound 9 on selected GPCRs**

**Assay (Agonist Effect)**	**% Of control agonist response**					**EC_{50}**
	**1st**	**2nd**	**Mean**	**S.E.M.**		
CCR3	0.0	-1.8	-0.9	0.9		6.4 nM
CCR4	-0.1	-0.1	-0.1	0		0.34 nM
CCR5	1.6	-4.6	-1.5	3.1		7.1 nM
CCR6	-0.5	-0.1	-0.3	0.2		0.31 nM
CCR7	-1.3	-2.3	-1.8	0.5		0.87 nM
CCR8	2.9	1.1	2.0	0.9		0.72 nM
CCR9	0.3	-0.1	0.1	0.2		2 nM
CCR10	-0.6	-1.2	-0.9	0.3		5 nM
CX3CR1	0.0	0.4	0.2	0.2		2.2 nM
CXCR1	-0.1	-1.4	-0.8	0.65		4.7 nM
CXCR3	0.5	0.0	0.3	0.25		2.3 nM
CXCR4	4.4	4.4	4.4	0		0.33 nM
CXCR5	2.0	0.4	1.2	0.8		3.8 nM
CXCR6	-0.8	-0.6	-0.7	0.1		1.6 nM
**Assay (Antagonist Effect)**	**Agonist Response (% of Control)**				**% Inhibition of control agonist response**	**IC_{50}**
	**1st**	**2nd**	**Mean**	**S.E.M.**		
CCR3	115.7	121.2	118.5	2.75	-0.9	8.9 nM
CCR4	60.7	88.2	74.4	13.75	-0.1	
CCR5	140.8	112.0	126.4	14.4	-1.5	
CCR6	105.3	105.9	105.6	0.3	-0.3	
CCR7	131.3	122.8	127.0	4.25	-1.8	
CCR8	116.9	80.2	98.5	18.35	2.0	
CCR9	108.5	95.2	101.9	6.65	0.1	
CCR10	85.4	90.0	87.7	2.3	-0.9	
CX3CR1	109.5	105.4	107.4	2.05	0.2	
CXCR1	87.1	105.5	96.3	9.2	-0.8	
CXCR3	102.6	102.1	102.3	0.25	0.3	
CXCR4	85.6	70.8	78.2	7.4	4.4	0.15 µM
CXCR5	103.3	99.7	101.5	1.8	1.2	
CXCR6	104.6	103.1	103.8	0.75	-0.7	

Cellular agonist effect was calculated as a % of control response to a known reference agonist for each target; cellular antagonist effect was calculated as a % inhibition of control reference agonist response for each target. Results showing an inhibition or stimulation higher than 50% are considered to represent significant effects of the test compounds. Number of replicates: 2. Reference compounds: CCR3: eotaxin 2 for agonist effect, J113863 for antagonist effect; CCR4: TARC; CCR5: MIP-1α; CCR6: MIP-3α; CCR7: MIP-3α; CCR8: I-309; CCR9: CCL25; CCR10: CTACK/CCL27; CX3CR1: CX3CL1; CXCR1: rhIL-8; CXCR3 :I-TAC; CXCR4: SDF-1α for agonist effect, MIP-II for antagonist effect; CXCR5: CXCL13/BLC/BCA-1; CXCR6: CXCL16.
